# FOLFIRINOX or Gemcitabine-based Chemotherapy for Borderline Resectable and Locally Advanced Pancreatic Cancer: A Multi-institutional, Patient-Level, Meta-analysis and Systematic Review

**DOI:** 10.1245/s10434-023-13353-2

**Published:** 2023-04-05

**Authors:** Dilmurodjon Eshmuminov, Botirjon Aminjonov, Russell F. Palm, Giuseppe Malleo, Ryan K. Schmocker, Raëf Abdallah, Changhoon Yoo, Walid L. Shaib, Marcel André Schneider, Elena Rangelova, Yoo Jin Choi, Hongbeom Kim, J. Bart Rose, Sameer Patel, Gregory C. Wilson, Sarah Maloney, Lea Timmermann, Klaus Sahora, Fabian Rössler, Víctor Lopez-Lopez, Emanuel Boyer, Laura Maggino, Thomas Malinka, Jeong Youp Park, Matthew H. G. Katz, Laura Prakash, Syed A. Ahmad, Scott Helton, Jin-Young Jang, Sarah E. Hoffe, Roberto Salvia, Julien Taieb, Jin He, Pierre-Alain Clavien, Ulrike Held, Kuno Lehmann

**Affiliations:** 1 Department of Surgery and Transplantation, University Hospital Zurich and University of Zurich, Zurich, Switzerland; 2 Department of Radiation Oncology, H. Lee Moffitt Cancer Center and Research Institute, Tampa, Florida USA; 3 Unit of General and Pancreatic Surgery. Department of Surgery, Dentistry, Paediatrics and Gynaecology, University of Verona, Verona, Italy; 4 Department of Surgery, The Division of Hepatobiliary and Pancreatic Surgery, Johns Hopkins Hospital, Baltimore, MD USA; 5 Department of Surgery, University of Tennessee Graduate School of Medicine, Knoxville, TN USA; 6 Hepatogastroenterology and Gastrointestinal Oncology Department, Hôpital Européen Georges-Pompidou, AGEO (Association des Gastro-Enterologues Oncologues), Université de Paris, SIRIC CARPEM, Paris, France; 7 Department of Oncology, Asan Medical Center, University of Ulsan College of Medicine, Seoul, Korea; 8 Department of Hematology and Oncology, Winship Cancer Institute, Emory University School of Medicine, Atlanta, GA USA; 9 Department of Upper Gastrointestinal Diseases, Karolinska University Hospital and Department of Clinical Science, Intervention, and Technology (CLINTEC) at Karolinska Institute, Stockholm, Sweden; 10 Department of Surgery, The Institute of Clinical Sciences, Sahlgrenska Academy, University of Gothenburg, Gothenburg, Sweden; 11 Department of Surgery, Seoul National University College of Medicine, Seoul National University, 28 Yongon-dong, Chongno-gu, Seoul, 110-744 Korea; 12 Division of Surgical Oncology, Pancreatobiliary Disease Center at UAB, The University of Alabama at Birmingham, Birmingham, USA; 13 Division of Surgical Oncology, Department of Surgery, University of Cincinnati, Cincinnati, OH USA; 14 Department of Oncology, Royal North Shore Hospital, Sydney, NSW Australia; 15 Department of Surgery, Charité – Freie Universität Berlin, Humboldt-Universität zu Berlin and Berlin Institute of Health, Berlin, Germany; 16 Departments of Surgery and Comprehensive Cancer Center, University of Vienna, Medical University of Vienna, Vienna, Austria; 17 Department of General, Visceral and Transplantation Surgery, Clinic and University Hospital Virgen de la Arrixaca, IMIB-ARRIXACA, Murcia, Spain; 18 University of South Florida School of Medicine, Tampa, FL USA; 19 Division of Gastroenterology, Department of Internal Medicine, Severance Hospital, Yonsei University College of Medicine, Seoul, Korea; 20 University of Texas MD Anderson Cancer Center, Houston, TX USA; 21 Section of General, Thoracic and Vascular Surgery, Department of Surgery, Virginia Mason Medical Center, Seattle, WA USA; 22 Department of Biostatistics at Epidemiology, Biostatistics and Prevention Institute, University of Zurich, Hirschengraben 84, 8001 Zurich, Switzerland

## Abstract

**Background:**

Pancreatic cancer often presents as locally advanced (LAPC) or borderline resectable (BRPC). Neoadjuvant systemic therapy is recommended as initial treatment. It is currently unclear what chemotherapy should be preferred for patients with BRPC or LAPC.

**Methods:**

We performed a systematic review and multi-institutional meta-analysis of patient-level data regarding the use of initial systemic therapy for BRPC and LAPC. Outcomes were reported separately for tumor entity and by chemotherapy regimen including FOLFIRINOX (FIO) or gemcitabine-based.

**Results:**

A total of 23 studies comprising 2930 patients were analyzed for overall survival (OS) calculated from the beginning of systemic treatment. OS for patients with BRPC was 22.0 months with FIO, 16.9 months with gemcitabine/nab-paclitaxel (Gem/nab), 21.6 months with gemcitabine/cisplatin or oxaliplatin or docetaxel or capecitabine (GemX), and 10 months with gemcitabine monotherapy (Gem-mono) (*p* < 0.0001). In patients with LAPC, OS also was higher with FIO (17.1 months) compared with Gem/nab (12.5 months), GemX (12.3 months), and Gem-mono (9.4 months; *p* < 0.0001). This difference was driven by the patients who did not undergo surgery, where FIO was superior to other regimens. The resection rates for patients with BRPC were 0.55 for gemcitabine-based chemotherapy and 0.53 with FIO. In patients with LAPC, resection rates were 0.19 with Gemcitabine and 0.28 with FIO. In resected patients, OS for patients with BRPC was 32.9 months with FIO and not different compared to Gem/nab, (28.6 months, *p* = 0.285), GemX (38.8 months, *p* = 0.1), or Gem-mono (23.1 months, *p* = 0.083). A similar trend was observed in resected patients converted from LAPC.

**Conclusions:**

In patients with BRPC or LAPC, primary treatment with FOLFIRINOX compared with Gemcitabine-based chemotherapy appears to provide a survival benefit for patients that are ultimately unresectable. For patients that undergo surgical resection, outcomes are similar between GEM+ and FOLFIRINOX when delivered in the neoadjuvant setting.

**Supplementary Information:**

The online version contains supplementary material available at 10.1245/s10434-023-13353-2.

Pancreatic ductal adenocarcinoma remains among the leading causes of cancer-related mortality.^1–3^ Most patients present with an advanced stage of disease, e.g., distant metastasis, or locally advanced (LAPC) or borderline resectable (BRPC) pancreatic cancer, ultimately resulting in a dismal 10-15% upfront resection rate and poor 5-year survival.^2^ In patients with BRPC or LAPC, primary chemotherapy with or without chemoradiation, hypofractionated radiotherapy, or stereotactic body radiotherapy has become increasingly utilized for local downstaging to enable a potentially curable resection. To this end, FOLFIRINOX (FIO) and Gemcitabine-based regimens (GEM+) are commonly used for primary treatment of pancreatic cancer.^4–6^ Generally, neoadjuvant FIO has been favored based on extrapolation of favorable outcomes in the adjuvant setting; however, this regimen carries a significant toxicity burden limiting use to patients with an excellent performance status.^7,8^ Gemcitabine continues to be used for its moderate side effect profile and is commonly paired with other therapies (GEM+) with increased biological activity.^6–9^ While PRODIGE 24 favored FIO over gemcitabine monotherapy in the adjuvant setting, these findings have yet to be demonstrated in the neoadjuvant setting with high level data.^6–9^ Prospective series investigating this question are available, but often include a limited number of patients, and are usually highly heterogeneous due to the combination of patients with resectable tumors, BRPC, and LAPC, which limit the ability to assess the impact of the type of neoadjuvant chemotherapy on surgical resection rate, positive margin rate, and survival.^6,8,10,11^

Thus, the objective of this study was to summarize the current evidence regarding initial systemic treatment for LAPC and BRPC through a multi-institutional meta-analysis of patient-level data from high-volume pancreatic centers. The primary goal of our meta-analysis was to assess the impact of primary chemotherapy, FIO, or GEM+ regimens on survival rates of patients with BRPC and LAPC. Secondary outcomes included resection rates, R0 resection rates, and impact of radiotherapy.

## Methods

### Study Search and Selection

The systematic review and meta-analysis were conducted following the PRISMA guidelines.^12^ The review protocol was registered in the PROSPERO database.^13^ The databases of Medline, Scopus Embase, and Cochrane were systematically searched for relevant clinical studies in English by a dedicated librarian without time restrictions in May 2020. The complete search strategy was provided in Appendix 1. The reference list of included studies was crosschecked manually to identify additional studies. All clinical studies reporting patients with primary pancreatic cancer without distant metastasis, who received primary FIO or GEM+ regimens, were included. Change in treatment regimen after initial treatment was not an exclusion criterion. Case series with less than ten patients, conference abstracts, letters, reviews, study protocols, and other type of nonoriginal articles were excluded.

### Data Collection and Outcome Measures

After removal of duplicates, two reviewers (D.E. and B.A.) independently screened all articles for relevance. Decision of article inclusion in the meta-analysis was done after full-text assessment, and discrepancies between reviewers were resolved by discussion and consensus. The following data were collected for meta-analysis: institution, study design, study population, tumor type (LAPC and BRPC), chemotherapy regimen, radiotherapy application, resection rate, R0 resection rate, and overall and disease-free survival. If study design was not clearly stated otherwise, the study was classified as a retrospective study. For survival analysis, we obtained patient level data from the studies listed in Table 1. Patients with available survival data from the time of systemic treatment were considered for the “full treatment population,” regardless of surgery at later timepoints. Secondary outcomes included resection rates, receipt of radiotherapy, R0 resection rates, and progression-free survival. Progression-free survival was calculated from the start of primary treatment.

### Statistical Methods

The individual patient-level data analysis included available time-to-event data from participating centers. Median follow-up time was estimated with the reverse Kaplan-Meier method. Time-to-event outcomes were visualized with the Kaplan Meier method. Hazard ratios for the comparison of FOLFIRINOX versus gemcitabine-based regimens were estimated and reported with 95% confidence intervals (CI). Survival analysis also was reported separately for resected and nonresected patients. The analysis did not account for clustering within centers. A meta-analysis was performed with dichotomous outcomes on study level in single groups, noncomparative data.^14^ Due to the expected heterogeneity of included studies, the random effects meta-analysis results were considered most relevant. Single-group, noncomparative data were estimated from all included studies, and proportions were reported either for FOI or GEM+ arm. These results were reported with 95% CIs. These results were reported as risk ratios (RR) with 95% CI. The heterogeneity was evaluated according to Cochrane Handbook for Systematic Reviews of Interventions with I^2^ statistics.^15^ I^2^ statistics was interpreted as follows: 0-40% not relevant heterogeneity; 30-60% moderate heterogeneity; 50-90% substantial heterogeneity; 75-100% considerable heterogeneity. Two-sided *p*-values were calculated in all analyses. The statistical programming language R, version 4.0.3 was used for survival analyses.

### Role of the Funding Source

There was no funding for this systematic review with meta-analysis.

## Results Included Studies and Descriptive Data

**Fig. 1:**
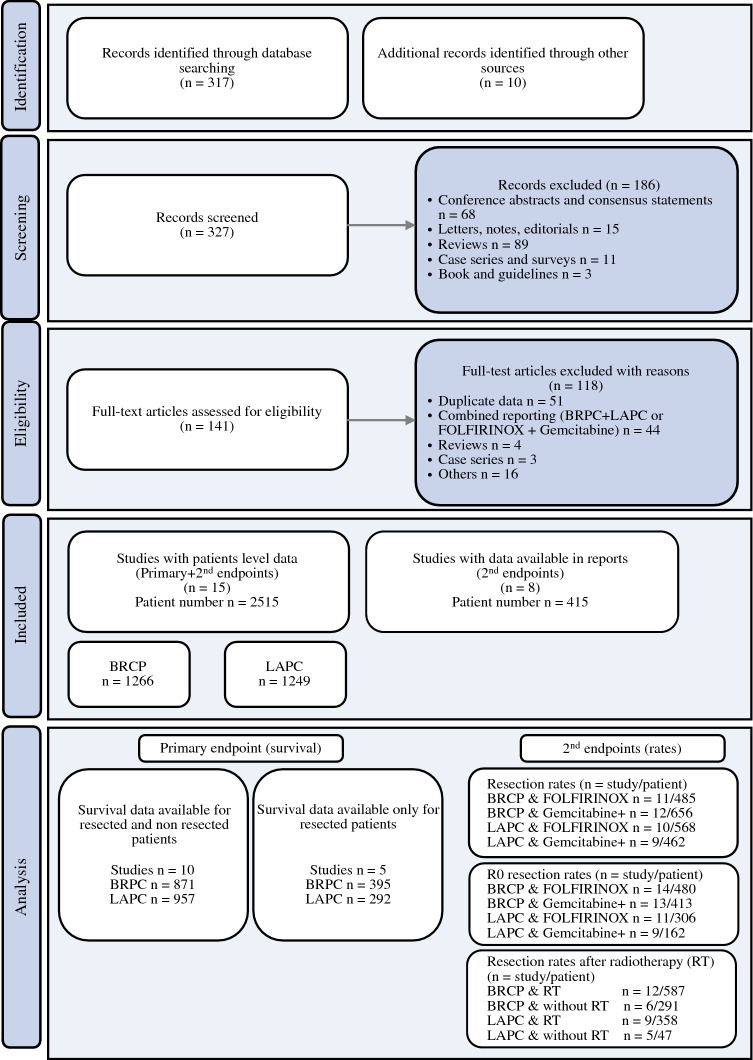
PRISMA flow chart

The systematic literature review identified 317 potential studies, and 10 studies were additionally identified manually. Of those, a total of 23 studies included 2,930 patients in the meta-analysis (Fig. 1). Of these publications, authors of 15 studies provided individual patient-level data for the 2,515 patients^6,10,16–28^ included in the survival analysis. Among the eight studies without patient level data, one is a randomized, controlled trial,^29^ four are prospective,^30–33^ and three retrospective observational studies.^34–36^ The majority of studies (n = 18) included patients from the start of systemic treatment. Five studies reported only patients undergoing resection. One of the main criteria for study exclusion during full-text assessment was combined reporting of BRPC together with LAPC, or FIO with GEM+. Therefore, separation of data for LAPC and BRPC was possible in all included studies reporting both tumor entities. For the definition of BRPC or LAPC, most studies used NCCN criteria (13 studies; Table 1). In ten studies, either GEM+ or FIO was used as systemic treatment; six studies report FIO only, in two of those studies a modified FIO (dose reduction to 75%) regimen was used, and seven studies report only GEM+. Radiotherapy (conventionally fractionated, hypofractionated, or stereotactic) was delivered in 19 studies.

### Overall Survival Analysis

**Fig. 2:**
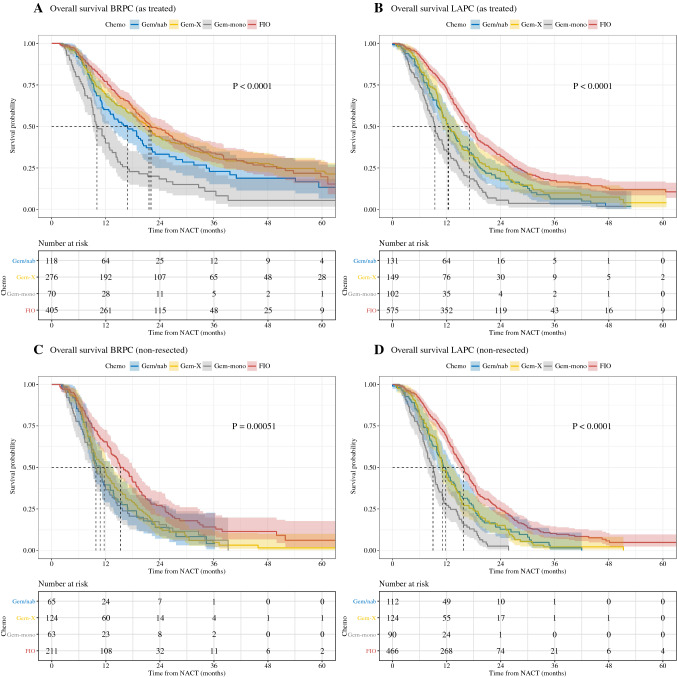
Overall survival with pooled, patient-level data for the intention for full treatment population (**A, B**) and non-resected patients (**C, D**) according to treatment regimen groups. *BRPC* borderline resectable pancreatic cancer; *LAPC* locally advanced pancreatic cancer; *FIO* FOLFIRINOX; *GEM/nab* gemcitabine/nab-paclitaxel; GemX gemcitabine/cisplatin or oxaliplatin or docetaxel or capecitabine; *Gem-mono* gemcitabine as monotherapy; *ITT* full treatment cohort

Overall survival was analyzed for patients “as treated,” based on diagnosis with BRPC and LAPC (Fig. 2). This analysis includes (n = 10) studies reporting on patient outcomes after the initialization of systemic treatment. Series reporting resected patients only (n = 5) were therefore excluded. Data were available for 869 BRPC patients with median follow-up of 44 months and for 957 LAPC patients with median follow-up of 37.9 months. We separately performed post-hoc overall survival analyses for the following groups: FIO, gemcitabine/nab-paclitaxel (Gem/nab), gemcitabine/cisplatin or oxaliplatin or docetaxel or capecitabine (GemX), and gemcitabine monotherapy (Gem-mono). For patients with BRPC, median overall survival for FIO was 22.0 months (95% CI 20.03–26.8), Gem/nab 16.9 months (95% CI 13.0–20.4), GemX 21.6 months (95% CI 18.7–24.0), and Gem-mono 10.1 months (95% CI 9.17–12.9; Fig. 2A). There was a statistically significant difference in overall survival across compared groups (*p* < 0.0001). In post-hoc analysis of FIO with other GEM+ regimens, a survival advantage for FIO was observed compared with Gem/nab (*p* = 0.012; hazard ratio of 0.718 (95% CI 0.555-0.929)) and Gem-mono (*p* < 0.0001; hazard ratio of 0.410 (95% CI 0.302-0.543)), while GemX disclosed the similar survival (*p* = 0.525; hazard ratio of 0.939 (95% CI 0.773-1.14)).

In patients with LAPC, the overall survival for FIO was 17.1 months (95% CI 16–18.5), compared with Gem/nab 12.5 months (95% CI 11.1–15.0), GemX 12.3 months (95% CI 11.1–14.9), and Gem-mono 9.4 months (95% CI 8.3–11.1; Fig. 2B). There was a statistically significant difference across compared groups (*p* < 0.0001). In post-hoc analysis, a survival advantage associated with FIO was observed compared with all GEM+ treatments (Gem/nab, GemX and Gem-mono *p* < 0.001 with hazard ratio of 0.618 (95% CI 0.500-0.763), 0.683 (95% CI 0.559-0.833), and 0.391 (95% CI 0.312-0.491) respectively).

### Survival in Unresected Patients

Patients without pancreatic tumor resection were analyzed separately as survival in pancreas cancer is heavily influenced by the ability to undergo resection. Data were available for 463 patients with BRPC and 792 patients with LAPC. In a separate analysis of nonresected patients, initially diagnosed as BRPC, overall survival for FIO was 15.3 months (95% CI 14–18), Gem/nab 10.8 months (95% CI 9.0–13.7), GemX 11.7 months (95% CI 9.7–13.4) and Gem-mono 9.8 months (95% CI 8.9–12.8). There was a statistically significant difference across compared groups (*p* = 0.0005) (Fig. 2C). In post hoc analysis of FIO with other GEM+ regimens, a survival advantage associated with FIO was observed (Gem/nab *p* = 0.003; hazard ratio of 0.620 (95% CI 0.452 to 0.850), GemX *p* = 0.002; hazard ratio of 0.676 (95% CI 0.528-0.865) and Gem-mono *p* = 0.001; hazard ratio of 0.600 (95% CI 0.442-0.816).

In patients with LAPC without resection, overall survival for FIO was 15.7 months (95% CI 14.7–17.0), Gem/nab 11.8 months (95% CI 10.0–13.8), GemX 11.1 months (95% CI 10.4–12.1) and Gem-mono 9.0 months (95% CI 7.7–10.0) (*p* < 0.0001; Fig. 2D). In post hoc analysis, FIO was associated with a survival advantage compared with all GEM+ regimens (Gem/nab, GemX, and Gem-mono *p* < 0.0001 with hazard ratio of 0.627 (95% CI 0.5-0.786), 0.622 (95% CI 0.504-0.768) and 0.361 (95% CI 0.283-0.460) respectively).

### Survival in Resected Patients

**Fig. 3:**
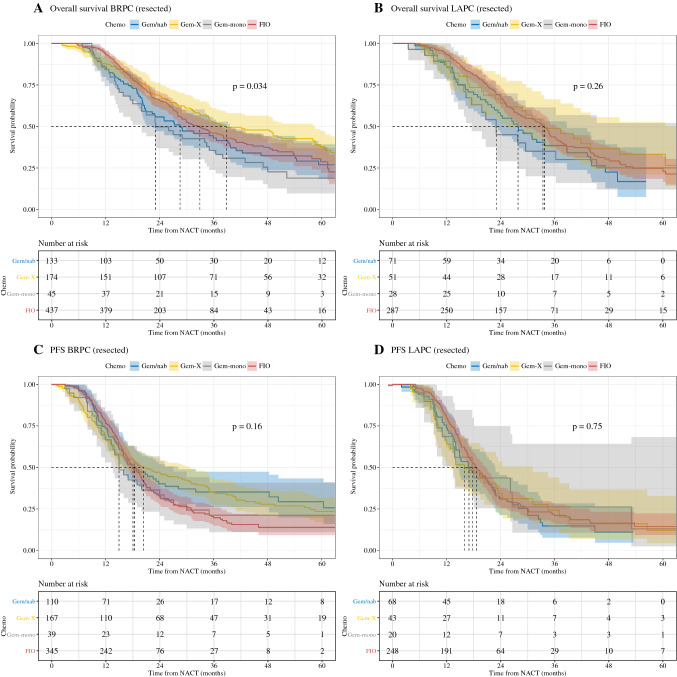
Overall (**A, B**) and progression-free (**C, D**) survival with pooled patient level data for resected patients, according to treatment regimen groups. *BRPC* borderline resectable pancreatic cancer; *LAPC* locally advanced pancreatic cancer; *FIO* FOLFIRINOX; *GEM/nab* gemcitabine/nab-paclitaxel; *GemX* gemcitabine/cisplatin or oxaliplatin or docetaxel or capecitabine; *Gem-mono* gemcitabine as monotherapy; *ITT* full treatment cohort

In analysis of all resected BRPC patients, patient level data were available for 789 patients with a median follow-up time of 46.4 months. In patients initially staged as BRPC and resected, median overall survival was 32.9 months (95% CI 28.9–38.2) for FIO, 28.6 months (95% CI 22.4–39.7) for Gem/nab, 38.8 months (95% CI 33.0–58.5) for GemX, and 23.1 months (95% CI 20.0–38.0) for Gem-mono (Fig. 3A). Overall, there was a statistically significant difference in compared groups (*p* = 0.034). However in post-hoc analysis, there was no statistically significant difference between FIO and other GEM+ regimens (Gem/nab *p* = 0.267; hazard ratio of 0.859 (95% CI 0.529-1.124), GemX *p* = 0.155; hazard ratio of 1.193 (95% CI 0.935-1.52), and Gem-mono *p* = 0.069; hazard ratio of 0.714 (95% CI 0.496-1.027)).

Of 437 patients with LAPC who underwent radical pancreatic surgery, the median follow-up was 44.5 months. Median overall survival was for FIO 33.4 months (95% CI 29.0–36.3), for Gem/nab 27.9 months (95% CI 22.7–38.8), for GemX 33.7 months (95% CI 25.3– 73.3), and for Gem-mono 23 months (lower bound of 95% CI 17.6) (*p* = 0.26; Fig. 3B). In post-hoc analysis of FIO with GEM+ regimens, no survival advantage of FIO was observed (Gem/nab *p* = 0.116; hazard ratio of 0.768 (95% CI 0.552 to 1.068), GemX *p* = 1.0 hazard ratio of 1 (95% CI 0.683-1.466), and Gem-mono *p* = 0.167; hazard ratio of 0.714 (95% CI 0.444-1.151).

There was no difference in disease-free survival by chemotherapy regimen for resected BRPC patients (*p* = 0.16, Fig. 3C) or resected LAPC patients (*p* = 0.75, Fig. 3D). Disease-free survival in BRCP was 18.1 months (95% CI 17.0–20.0) for FIO, 18.4 months (95% CI 15.9–28.2) for Gem/nab, 20.5 months (95% CI 15.4–30.9) for GemX, and 15 months (95% CI 13.3–27.1) for Gem-mono. Disease-free survival for patients with LAPC was 18.7 months (95% CI 17.0–20.8) for FIO, 17 months (95% CI 14.1–22.8) for Gem/nab, 16 months (95% CI 12.9–31.2) for GemX, and 17.7 months (lower bound of 95% CI 13.4) for Gem-mono.

### Meta-Analysis of Secondary Endpoints

**Fig. 4:**
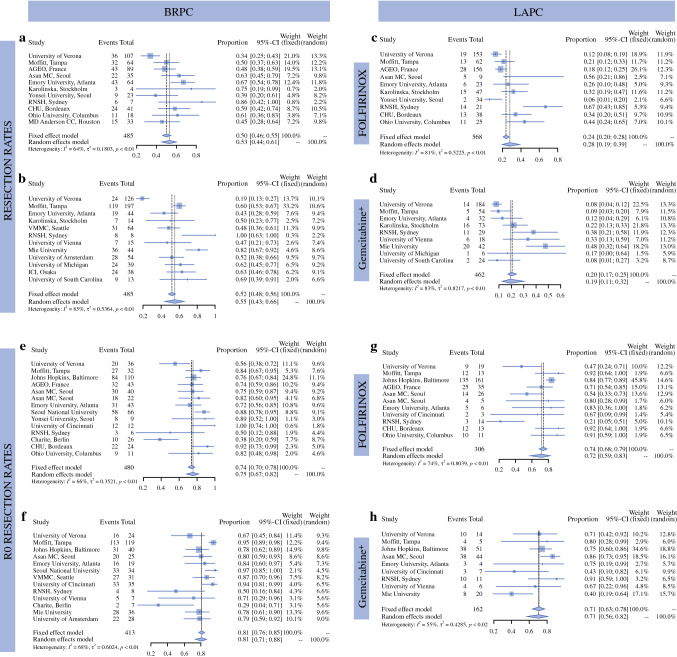
Meta-analysis of resection and R0-resection rates. **a, b:** Resection rates for patients with BRCP or LAPC after FOLFIRINOX or Gemcitabine+. **c, d:** Resection rates in patients with LAPC after FOLFIRINOX or Gemcitabine+. **e, f:** R0 resection rates in patients with BRCP after FOLFIRINOX or Gemcitabine+. **g, h:** R0 resection rates in patients with LAPC after FOLFIRINOX or Gemcitabine+. Overall, we observed a substantial heterogeneity (I^2^) in all analyses

Secondary analyses of overall resection rate, R0 resection rate, and resection rate after radiation therapy for patients with these reported endpoints: For patients with BRPC, the overall resection rates were 0.53 and 0.55, and R0 resection rates were 0.75 and 0.81 for FIO and GEM+ regimens, respectively. In patients with LAPC, overall resection rates were 0.28 and 0.19, and R0 resection rates were 0.72 and 0.71 for FIO and GEM+ regimens, respectively (Fig. 4).

Details regarding technique and dose of radiotherapy were lacking from most studies. Thus, we combined chemotherapy regimens and explored the value of radiotherapy in increasing the resection rate in the preoperative setting for BRPC and LAPC. In BRPC, resection rates with and without radiotherapy were 0.58 and 0.51. For LAPC, resection rates with and without radiotherapy were 0.24 and 0.21 (Supplementary Fig. 1).

## Discussion

This study is the largest, systematic review and multi-institutional, patient-level meta-analysis to examine the impact of neoadjuvant systemic treatment with FOLFIRINOX (FIO) or gemcitabine-based regimens (GEM+) in patients with BRPC or LAPC. Our data suggest the superiority of FIO over GEM+ in both the BRPC and LAPC patient population. However, this benefit is driven in patients who do not undergo surgery. In patients who ultimately undergo resection, the survival outcomes were similar. It may be argued that including patients who received gemcitabine, monotherapy may be considered a palliative treatment and multiagent chemotherapy should be favored whenever possible.

In the setting of primary, resectable pancreatic cancer, surgery followed by adjuvant chemotherapy remains the standard approach. However, neoadjuvant therapy is being increasingly utilized in this setting as evidenced by the increase number of clinical trials.^37^ Over the past decades, major advances in novel combination drug therapies and aggressive dose adjustments have significantly improved patient outcomes in the adjuvant setting. Dismal outcomes with 5-FU monotherapy was supplanted by significant survival improvements with the introduction of gemcitabine in 1997.^38^ Today, FOLFIRINOX and multidrug, gemcitabine-based regimens have shown benefits in the adjuvant and palliative settings and are now a standard of care.^7,39,40^ In contrast to primary, resectable pancreatic cancer, data suggest that patients with BRPC or LAPC have improved outcomes when treated with primary chemotherapy with or without radiotherapy.^29,41,42^ Based on the success of FOLFIRINOX and GEM+ in the palliative setting, these combination regimens are currently used as preoperative systemic treatment for BRPC and LAPC. There are two available meta-analysis with patient level data examining survival outcomes for FOLFIRINOX-based treatments for BRPC.^43,44^ This paucity of data has left significant knowledge gaps for high-level data for other important clinical outcomes (such as resectability and progression-free survival) as well as optimal treatment of patients with LAPC. A recent, multicenter, phase II study from Germany evaluated Gemcitabine/nab-Paclitaxel versus FOLFIRINOX in patients after initial treatment with two cycles of Gemcitabine/nab-Paclitaxel and found similar conversion and survival rates in both arms; however, this was not a direct comparison of these regimens.^45^ Comparison of the two regimens within two, single-institution, cohort studies did not favor either treatment strategy as both resulted in similar survival rates.^6,8^ In patients who do not undergo surgery, our results suggest improved survival rates for patients treated with FOLFIRINOX compared with gemcitabine-based regimens. However, this result was influenced by the outcome of nonresected patients. In patients who underwent resection, we did not observe a benefit for FOLFIRINOX neither in BRPC nor in LAPC. This finding suggests that surgical resection of the tumor remains a critical driver of survival, and the type of chemotherapy may be less important than the duration and sequencing of treatment. Clearly, conversion to surgical resectability also represents the biology of the tumor, implying a less aggressive or chemosensitive tumor.

To evaluate outcomes, particularly after surgery, it is critical to stratify by the local stage of the pancreatic tumor. During the past years, the concept of BRPC and LAPC evolved and is currently defined by the tumor involvement of the celiac and mesenteric arteries and the portal vein/superior mesenteric vein.^46^ It is important to mention that there is significant heterogeneity in the definitions of BRPC or LAPC across institutions.^47^ Moreover, combined reporting of LAPC and BRPC may significantly skew the results, depending on the number of included entities, which was the case in previous meta-analyses. The ability to analyze these patient groups separately is a primary strength of this study. Indeed, the resection rate in two previous meta-analyses differed from 28% to 68% due to the pooling of BRPC and LAPC patients one of these studies.^43,44^ The importance of the distinction between BRPC and LAPC is highlighted in this study with the observation that BRPC could be resected in approximately half of the patients after systemic chemotherapy, whereas less than a third of LAPC patients underwent definitive surgical resection after neoadjuvant treatment.

Despite advanced local stage, the R0 resection rates in BRPC and LAPC were high (0.71-0.81) and are well above a recently reported benchmark for pancreatoduodenectomy where R0 resection rates of at least 0.61 were proposed for primary resectable pancreatic cancer.^48^ This high R0 resection rate reflects the experience of the included centers and but also may belay the benefit of aggressive neoadjuvant treatment, because these high R0 rates were achieved despite more advanced disease.

Due to a lack of comparative data in our analysis, no definitive conclusion should be drawn regarding the effect of neoadjuvant or definitive radiotherapy, which is similar to limitations discussed in previous meta-analyses.^41,43^ There is significant heterogeneity in the treatment fields, biologically effective dose (BED), and fractionation between institutions in treatment of pancreatic cancer. Furthermore, the combination of different modalities in the neoadjuvant setting often precludes current interpretation of a distinct role of one technique over the other. For example, in the recently published, Dutch, PREOPANC-1 study, patients with BRPC were randomized between immediate surgery and primary moderately hypofractionated chemoradiotherapy with Gemcitabine.^29^ Preoperative chemoradiotherapy provided a significant overall survival benefit, but the added value of radiotherapy in addition to chemotherapy remains unclear. To this end, the LAP07 trial was unable to demonstrate a survival benefit in the addition of conventionally fractionated, low-BED radiotherapy to gemcitabine monotherapy for LAPC; however, there was a 14% improvement in local control.^49^ More recent evidence favors stereotactic radiotherapy as delivered in LAPC-1 after FOLFIRINOX, which demonstrated a survival benefit, but lacks a comparative group not treated with of radiotherapy.^50^ Finally, for unresected patients, ablative doses approaching 100 Gy BED may be associated with the best outcomes for LAPC,^51^ and new technology, such as MRI-guided, adaptive radiotherapy, may facilitate delivery of this treatment safely.^52^ The Alliance for Clinical Oncology Trial A021501compared mFOLFIRINOX with and without hypofractionated radiotherapy in patients with BRPC. The arm with hypofractionated radiotherapy disclosed 67% R1 resection rate, whereas the other arm 43%. This randomized, controlled trial concluded that mFOLFIRINOX alone is superior compared with mFOLFIRINOX plus hypofractionated radiotherapy in neoadjuvant setting. However, this randomized, controlled trial terminated prematurely and is ultimately underpowered to report the value of the addition of hypofractionated radiotherapy for patients with BRPC.^53–55^

We acknowledge the limitations of this meta-analysis. There is clearly a lack of prospectively collected, randomized data comparing FOLFIRINOX with gemcitabine-based regimens in patients with BRPC or LAPC. Therefore, the choice of the regimen is left to the physician.^43^ None of the included studies reported a decision to use either FOLFIRINOX or GEM+, which reflects the current, real-world dilemma. Due to a more favorable side-effect profile, Gemcitabine-based regimens may be chosen for patients with poor performance status, whereas patients with a higher performance status receive FOLFIRINOX. The number of chemotherapy cycles varied across studies; however, a previous meta-analysis demonstrated this may not have a major impact.^43^ Further the data on the rate of complications of systemic therapy, i.e., how many patients interrupt the therapy or need a modification of therapy were not available. Another bias is the role of radiotherapy. Not all included studies performed radiotherapy in the multimodal management of pancreatic cancer in the neoadjuvant setting. The literature search was conducted 18 months ago. Since publications, some further studies were conducted and not included in this analysis.^55–57^ It is important to highlight that heterogeneity exists between institutional and international committee definitions of BRCP and LAPC.^46^ Despite these limitations, our ability to examine patient-level data, examine patients who were and were not resected, and the ability to analyze BRCP and LAPC patients separately offers significant value to this important clinical question. Finally, we were able to offer subanalyses on the variety of combinations of Gemcitabine-based chemotherapy regimens, which further improves the real-world applicability of our findings.

## Conclusions

In the setting of BRPC and LAPC, our data suggest that FOLFIRINOX may be preferred for patients with good performance status due to the survival benefit in patients who are ultimately not resected. Combination therapy with gemcitabine can be considered as a reasonable alternative to FOLFIRINOX, particularly in patients who have a less robust performance status or who are expected to ultimately undergo definitive surgical resection. Prospective data are needed to clarify the optimal timing, duration, and type of chemotherapy regimen as well as the appropriate use of radiotherapy in neoadjuvant or definitive treatment for the heterogeneous patient population that comprises BRPC and LAPC.

## Supplementary Information

Below is the link to the electronic supplementary material.

Supplementary file1 (JPG 254 kb)

Supplementary file2 (DOCX 103 kb)
