# Supplementary figures and images for: FOLFIRINOX or Gemcitabine-based Chemotherapy for Borderline Resectable and Locally Advanced Pancreatic Cancer: A Multi-institutional, Patient-Level, Meta-analysis and Systematic Review
Source: Ann Surg Oncol. 2023 Apr 5;30(7):4417–28. doi: 10.1245/s10434-023-13353-2 (PMC10250524; doi:10.1245/s10434-023-13353-2)

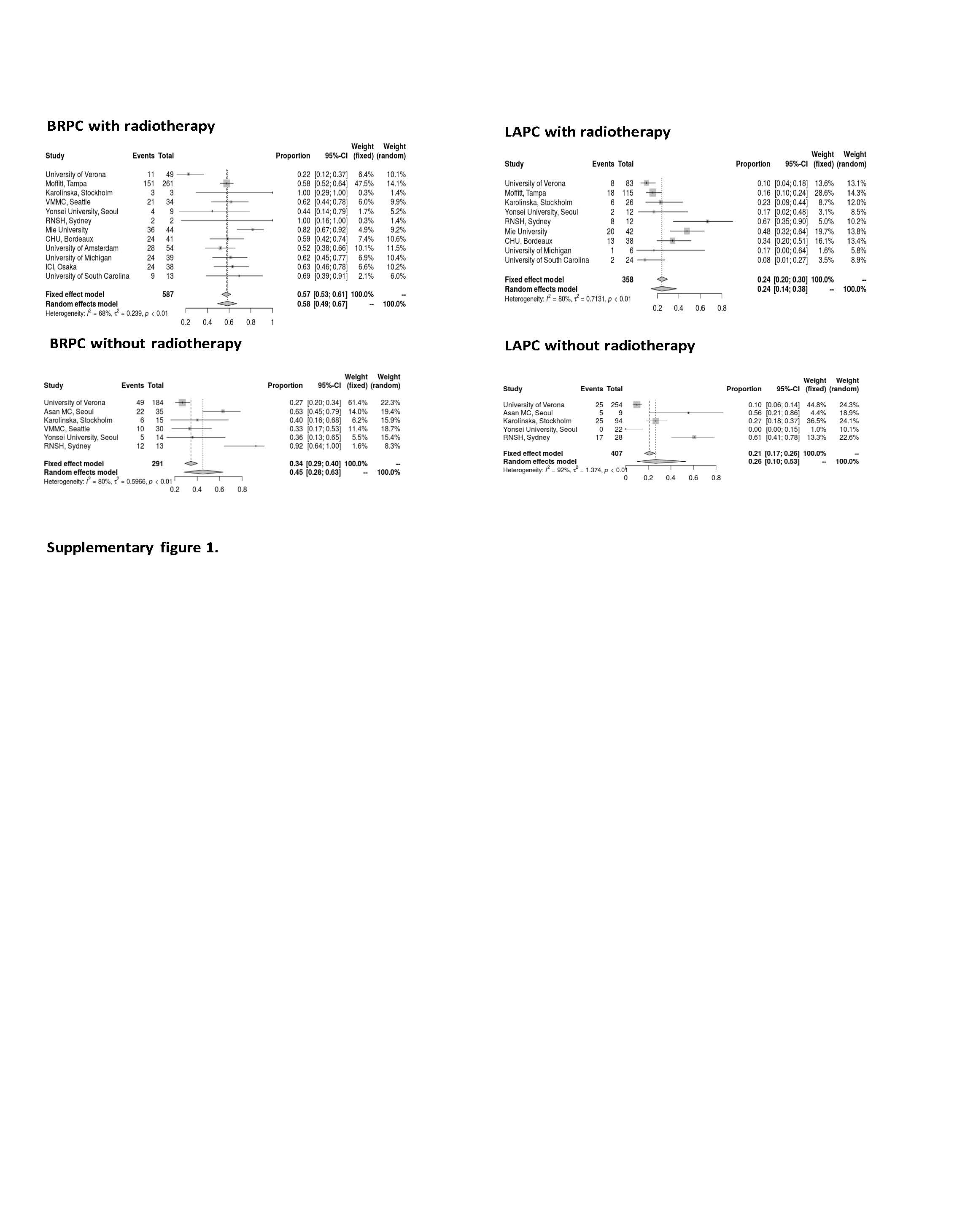

Supplement: Supplementary file 1 — Supplementary file1 (JPG 254 kb) [file 10434_2023_13353_MOESM1_ESM.jpg]
