# Supplementary material for: FOLFIRINOX or Gemcitabine-based Chemotherapy for Borderline Resectable and Locally Advanced Pancreatic Cancer: A Multi-institutional, Patient-Level, Meta-analysis and Systematic Review
Source: Ann Surg Oncol. 2023 Apr 5;30(7):4417–28. doi: 10.1245/s10434-023-13353-2 (PMC10250524; doi:10.1245/s10434-023-13353-2)
Supplement: Supplementary file 2 — Supplementary file2 (DOCX 103 kb) [file 10434_2023_13353_MOESM2_ESM.docx]

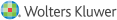

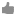

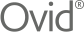


[Support & Training](http://www.ovid.com/support-training.html) [Feedback](https://www.surveymonkey.com/r/55CCV3R) Close

Database(s): **Ovid MEDLINE(R) and Epub Ahead of Print, In-Process & Other Non-Indexed Citations and Daily** 1946 to May 06, 2020

Search Strategy:

| **#** | **Searches** | **Results** |
| --- | --- | --- |
| 1 | borderline.mp. and (exp Pancreatic Neoplasms/ or ((pancreatic or pancreas) adj3 (cancer or neoplasm* or carcinoma* or adenocarcinoma* or adenoma*  or tumor* or tumour*)).ti,ab.) | 1183 |
| 2 | folfirinox.mp. or ((exp Leucovorin/ or ("5 formyl 5, 6, 7, 8 tetrahydrofolic acid" or "5 formyltetrahydrofolate" or "5 formyltetrahydrofolic acid" or "5 formyltetrahydropteroylglutamic acid" or "folinic acid" or "folinic acid sf" or "folinic acid sf rescue" or "formyltetrahydrofolate" or "formyltetrahydrofolates" or "formyltetrahydropteroylglutamic acid" or "lencovorin" or "leucovorin" or "leukovoran" or "leukovorin" or "n formyltetrahydrofolic acid" or "n5 formyl 5, 6, 7, 8 tetrahydropteroylglutamic acid" or "n5 formyl tetrahydrofolic acid" or "n5 formyltetrahydrofolate" or "n5 formyltetrahydrofolic acid").ti,ab.) and (exp Fluorouracil/ or ("2, 4 dioxo 5 fluoropyrimidine" or "5 fluoro 2, 4 pyrimidinedione" or "5 fluoro uracil" or "5 fluoropyrimidine 2, 4 dione" or "5 fluorouracil" or "5 fluoruracil" or "5 fu" or "accusite" or "actino-hermal" or "adrucil" or "agicil" or "carac" or "cinkef u" or "cinkef-u" or "effluderm" or "efudex" or "efudix" or "efurix" or "eurofluor (drug)" or "f6627" or "fivoflu" or "fluoro uracil" or "fluoroblastin" or "fluoroplex" or "fluorouracil" or "fluorouracil 5" or "fluorouracil sodium" or "fluorouracile" or "fluorouracilo" or "fluoruracil" or "fluouracil" or "fluoxan" or "flurablastin" or "fluracedyl" or "fluracil" or "fluracilium" or "fluril" or "fluro uracil" or "fluroblastin" or "fluroblastine" or "ifacil" or "nsc 18913" or "nsc 19893" or "nsc18913" or "nsc19893" or "oncofu" or "ribofluor" or "ro 2 9757" or "ro 2-9757" or "ro2 9757" or "ro2-9757" or "tolak" or "uflahex" or "uraciflor" or "utoral").ti,ab.) and (exp Irinotecan/ or ("7 ethyl 10 (4 piperidinopiperidinocarbonyloxy) camptothecin" or "calmtop" or "campto" or "camptosar" or "camptothecin, 7 ethyl 10 (4 piperidinopiperidinocarbonyloxy)" or "cpt 11" or "cpt11" or "irinotecan" or "irinotecan hydrochloride" or "irinotecan hydrochloride trihydrate" or "irinotel" or "topotecin").ti,ab.) and (exp Oxaliplatin/ or ("axiplatin" or "bendaplatin" or "crisapla" or "croloxat" or "dacotin" or "dacplat" or "ebeoxal" or "elatofen" or "eloxatin" or "eloxatine" or "elplat" or "euroxaliplatin" or "geneplatin" or "gessedil" or "heloxatin" or "lipoxal" or "mbp 426" or "mbp426" or "medoxa" or "oksaliplatin" or "oksaliplatina" or "oplat" or "oxalato 1, 2 cyclohexanediamine platinum" or "oxalatoplatinum" or "oxalatplatin" or "oxali" or "oxalip" or "oxaliplan" or "oxaliplatin" or "oxaliplatina" or "oxaliplatine" or "oxaliplatino" or "oxaliplatinum" or "oxaliprol" or "oxaliquid" or "oxalisan" or "oxalisin" or "oxalizor" or "oxaltic" or "oxaltina" or "oxamed (oxaliplatin)" or "oxaplamyl" or "oxaviatin" or "platinum 1, 2 cyclohexanediamine oxalate" or "platinum 1, 2 diaminocyclohexane oxalate" or "platinum oxalate 1, 2 diaminocyclohexane" or "platinum trans (oxalato) (1, 2 diaminocyclohexane)" or "platox" or "plaxitin" or "rectoxal" or "riboxatin" or  "rp 54780" or "rp54780" or "sinoxal" or "sr 96669" or "sr96669" or "transplastin" or "velminox" or "xaliplat" or "xoplan").ti,ab.)) | 1834 |
| 3 | (exp Paclitaxel/ or ("abi 007" or "abi007" or "abraxane" or "albumin bound paclitaxel" or "albumin-bound paclitaxel" or "anzatax" or "apealea" or "asotax" or "biotax" or "bms 181339" or "bms181339" or "bmy 45622" or "bmy45622" or "bristaxol" or "britaxol" or "coroxane" or "dts 301" or "dts301" or "endotag- 1" or "formoxol" or "genexol" or "genexol pm" or "hunxol" or "ifaxol" or "infinnium" or "intaxel" or "mbt 0206" or "mbt0206" or "medixel" or "mitotax" or "nab paclitaxel" or "nanoparticle albumin bound paclitaxel" or "nsc 125973" or "nsc 673089" or "nsc125973" or "nsc673089" or "oas pac 100" or "oaspac100" or "oncogel" or "onxol" or "pacitaxel" or "paclitaxel" or "paclitaxel nab" or "pacxel" or "padexol" or "parexel" or "paxceed" or "paxene" or "paxus" or "pazenir" or "praxel" or "sb 05 (terpenoid)" or "sb05 (terpenoid)" or "taxocris" or "taxol" or "taxus (drug)" or "taycovit" or "yewtaxan").ti,ab.) and ("2` deoxy 2`, 2` difluorocytidine" or "2`, 2` difluorodeoxycytidine" or "d 07001" or "d07001" or "difluorodeoxycytidine" or "ff 10832" or "ff10832" or "gembin" or "gemci-cell" or "gemcisela" or "gemcitabin" or "gemcitabina" or "gemcitabine" or "gemcitabine hydrochloride" or "gemcitabinum" or "gemcite" or "gemcitina" or "gemcitom" or "gemedac" or "gemkabi" or "gemliquid (drug)" or "gemsol (drug)" or "gemstad" or "gemstada" or "gemtro" or "gemzar" or  "getmisi" or "gitrabin" or "infugem" or "ly 188011" or "ly188011" or "ribozar").ti,ab. | 2619 |
| 4 | 1 and (2 or 3) | 139 |
| 5 | limit 4 to english language | 130 |

1. Increased Rate of Complete Pathologic Response After Neoadjuvant **FOLFIRINOX** for BRCA Mutation Carriers with **Borderline** Resectable

**Pancreatic Cancer**.

Golan T; Barenboim A; Lahat G; Nachmany I; Goykhman Y; Shacham-Shmueli E; Halpern N; Brazowski E; Geva R; Wolf I; Goldes Y; Ben-Haim M; Klausner JM; Lubezky N.

*Annals of Surgical Oncology. 2020 Apr 20. [Journal Article]*

**UI:** 32314163

**Authors Full Name**

Golan, Talia; Barenboim, Alex; Lahat, Guy; Nachmany, Ido; Goykhman, Yacov; Shacham-Shmueli, Einat; Halpern, Naama; Brazowski, Eli; Geva, Ravit; Wolf, Ido; Goldes, Yuri; Ben-Haim, Menahem; Klausner, Joseph M; Lubezky, Nir.


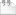
[Cite](http://ovidsp.dc2.ovid.com/sp-4.05.0b/ovidweb.cgi?&S=NABGFPCEPGEBMEOJIPBKOHOGCBHNAA00&Get%2BCitation=S.sh.88%7c1)


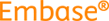


Embase Session Results (7 May 2020)

No. Query Results

#6 #1 AND (#2 OR #3) NOT [conference abstract]/lim AND [english]/lim 252

#5 #1 AND (#2 OR #3) NOT [conference abstract]/lim 268

#4 #1 AND (#2 OR #3) 552

('paclitaxel'/exp OR 'abi 007':ti,ab OR 'abi007':ti,ab OR 'abraxane':ti,ab OR 'albumin bound paclitaxel':ti,ab OR 'albumin-bound paclitaxel':ti,ab OR 'anzatax':ti,ab OR 'apealea':ti,ab OR 'asotax':ti,ab OR 'biotax':ti,ab OR 'bms 181339':ti,ab OR 'bms181339':ti,ab OR 'bmy 45622':ti,ab OR 'bmy45622':ti,ab OR 'bristaxol':ti,ab OR 'britaxol':ti,ab OR 'coroxane':ti,ab OR 'dts 301':ti,ab OR 'dts301':ti,ab OR 'endotag-1':ti,ab OR 'formoxol':ti,ab OR 'genexol':ti,ab OR 'genexol pm':ti,ab OR 'hunxol':ti,ab OR 'ifaxol':ti,ab OR 'infinnium':ti,ab OR 'intaxel':ti,ab OR 'mbt 0206':ti,ab OR 'mbt0206':ti,ab OR 'medixel':ti,ab OR 'mitotax':ti,ab OR 'nab paclitaxel':ti,ab OR 'nanoparticle albumin bound paclitaxel':ti,ab OR 'nsc 125973':ti,ab OR 'nsc 673089':ti,ab OR 'nsc125973':ti,ab OR 'nsc673089':ti,ab OR 'oas pac 100':ti,ab OR 'oaspac100':ti,ab OR 'oncogel':ti,ab OR 'onxol':ti,ab OR 'pacitaxel':ti,ab OR 'paclitaxel':ti,ab OR 'paclitaxel nab':ti,ab OR 'pacxel':ti,ab OR 'padexol':ti,ab OR

#3 'parexel':ti,ab OR 'paxceed':ti,ab OR 'paxene':ti,ab OR 'paxus':ti,ab OR 'pazenir':ti,ab OR 'praxel':ti,ab OR 'sb 05 (terpenoid)':ti,ab OR 'sb05 (terpenoid)':ti,ab OR 'taxocris':ti,ab OR 'taxol':ti,ab OR 'taxus (drug)':ti,ab OR 'taycovit':ti,ab OR 'yewtaxan':ti,ab) AND ('gemcitabine'/exp OR '2` deoxy 2`, 2` difluorocytidine':ti,ab OR '2`, 2` difluorodeoxycytidine':ti,ab OR 'd 07001':ti,ab OR 'd07001':ti,ab OR 'difluorodeoxycytidine':ti,ab OR 'ff 10832':ti,ab OR 'ff10832':ti,ab OR 'gembin':ti,ab OR 'gemci-cell':ti,ab OR 'gemcisela':ti,ab OR 'gemcitabin':ti,ab OR 'gemcitabina':ti,ab OR 'gemcitabine':ti,ab OR 'gemcitabine hydrochloride':ti,ab OR 'gemcitabinum':ti,ab OR 'gemcite':ti,ab OR 'gemcitina':ti,ab OR 'gemcitom':ti,ab OR 'gemedac':ti,ab OR 'gemkabi':ti,ab OR 'gemliquid (drug)':ti,ab OR 'gemsol (drug)':ti,ab OR 'gemstad':ti,ab OR 'gemstada':ti,ab OR 'gemtro':ti,ab OR 'gemzar':ti,ab OR 'getmisi':ti,ab OR 'gitrabin':ti,ab OR 'infugem':ti,ab OR 'ly 188011':ti,ab OR 'ly188011':ti,ab OR 'ribozar':ti,ab)

'folfirinox'/exp OR folfirinox:ti,ab,kw OR (('folinic acid'/exp OR '5 formyl 5, 6, 7, 8 tetrahydrofolic acid':ti,ab OR

'5 formyltetrahydrofolate':ti,ab OR '5 formyltetrahydrofolic acid':ti,ab OR '5 formyltetrahydropteroylglutamic acid':ti,ab OR 'folinic acid':ti,ab OR 'folinic acid sf':ti,ab OR 'folinic acid sf rescue':ti,ab OR 'formyltetrahydrofolate':ti,ab OR 'formyltetrahydrofolates':ti,ab OR 'formyltetrahydropteroylglutamic acid':ti,ab OR 'lencovorin':ti,ab OR 'leucovorin':ti,ab OR 'leukovoran':ti,ab OR 'leukovorin':ti,ab OR 'n formyltetrahydrofolic acid':ti,ab OR 'n5 formyl 5, 6, 7, 8 tetrahydropteroylglutamic acid':ti,ab OR 'n5 formyl tetrahydrofolic acid':ti,ab OR 'n5 formyltetrahydrofolate':ti,ab OR 'n5 formyltetrahydrofolic acid':ti,ab) AND ('fluorouracil'/exp OR '2, 4 dioxo 5 fluoropyrimidine':ti,ab OR '5 fluoro 2, 4 pyrimidinedione':ti,ab OR '5 fluoro

uracil':ti,ab OR '5 fluoropyrimidine 2, 4 dione':ti,ab OR '5 fluorouracil':ti,ab OR '5 fluoruracil':ti,ab OR '5 fu':ti,ab OR 'accusite':ti,ab OR 'actino-hermal':ti,ab OR 'adrucil':ti,ab OR 'agicil':ti,ab OR 'carac':ti,ab OR 'cinkef u':ti,ab OR 'cinkef-u':ti,ab OR 'effluderm':ti,ab OR 'efudex':ti,ab OR 'efudix':ti,ab OR 'efurix':ti,ab OR 'eurofluor (drug)':ti,ab OR 'f6627':ti,ab OR 'fivoflu':ti,ab OR 'fluoro uracil':ti,ab OR 'fluoroblastin':ti,ab OR 'fluoroplex':ti,ab OR 'fluorouracil':ti,ab OR 'fluorouracil 5':ti,ab OR 'fluorouracil sodium':ti,ab OR 'fluorouracile':ti,ab OR 'fluorouracilo':ti,ab OR 'fluoruracil':ti,ab OR 'fluouracil':ti,ab OR 'fluoxan':ti,ab OR 'flurablastin':ti,ab OR 'fluracedyl':ti,ab OR 'fluracil':ti,ab OR 'fluracilium':ti,ab OR 'fluril':ti,ab OR 'fluro uracil':ti,ab OR 'fluroblastin':ti,ab OR 'fluroblastine':ti,ab OR 'ifacil':ti,ab OR 'nsc 18913':ti,ab OR 'nsc 19893':ti,ab OR 'nsc18913':ti,ab OR 'nsc19893':ti,ab OR 'oncofu':ti,ab OR 'ribofluor':ti,ab OR 'ro 2 9757':ti,ab OR 'ro 2-9757':ti,ab OR 'ro2

#2 9757':ti,ab OR 'ro2-9757':ti,ab OR 'tolak':ti,ab OR 'uflahex':ti,ab OR 'uraciflor':ti,ab OR 'utoral':ti,ab) AND ('irinotecan'/exp OR '7 ethyl 10 (4 piperidinopiperidinocarbonyloxy) camptothecin':ti,ab OR 'calmtop':ti,ab OR 'campto':ti,ab OR 'camptosar':ti,ab OR 'camptothecin, 7 ethyl 10 (4 piperidinopiperidinocarbonyloxy)':ti,ab OR 'cpt 11':ti,ab OR 'cpt11':ti,ab OR 'irinotecan':ti,ab OR 'irinotecan hydrochloride':ti,ab OR 'irinotecan hydrochloride trihydrate':ti,ab OR 'irinotel':ti,ab OR 'topotecin':ti,ab) AND ('oxaliplatin'/exp OR 'axiplatin':ti,ab OR 'bendaplatin':ti,ab OR 'crisapla':ti,ab OR 'croloxat':ti,ab OR 'dacotin':ti,ab OR 'dacplat':ti,ab OR 'ebeoxal':ti,ab OR 'elatofen':ti,ab OR 'eloxatin':ti,ab OR 'eloxatine':ti,ab OR 'elplat':ti,ab OR 'euroxaliplatin':ti,ab OR 'geneplatin':ti,ab OR 'gessedil':ti,ab OR 'heloxatin':ti,ab OR 'lipoxal':ti,ab OR 'mbp 426':ti,ab OR 'mbp426':ti,ab OR 'medoxa':ti,ab OR 'oksaliplatin':ti,ab OR 'oksaliplatina':ti,ab OR 'oplat':ti,ab OR 'oxalato 1, 2 cyclohexanediamine platinum':ti,ab OR 'oxalatoplatinum':ti,ab OR 'oxalatplatin':ti,ab OR 'oxali':ti,ab OR 'oxalip':ti,ab OR 'oxaliplan':ti,ab OR 'oxaliplatin':ti,ab OR 'oxaliplatina':ti,ab OR 'oxaliplatine':ti,ab OR 'oxaliplatino':ti,ab OR 'oxaliplatinum':ti,ab OR 'oxaliprol':ti,ab OR 'oxaliquid':ti,ab OR 'oxalisan':ti,ab OR 'oxalisin':ti,ab OR 'oxalizor':ti,ab OR 'oxaltic':ti,ab OR 'oxaltina':ti,ab OR 'oxamed (oxaliplatin)':ti,ab OR 'oxaplamyl':ti,ab OR 'oxaviatin':ti,ab OR 'platinum 1, 2 cyclohexanediamine oxalate':ti,ab OR 'platinum 1, 2 diaminocyclohexane oxalate':ti,ab OR 'platinum oxalate 1, 2 diaminocyclohexane':ti,ab OR 'platinum trans (oxalato) (1, 2 diaminocyclohexane)':ti,ab OR 'platox':ti,ab OR 'plaxitin':ti,ab OR 'rectoxal':ti,ab OR 'riboxatin':ti,ab OR 'rp 54780':ti,ab OR 'rp54780':ti,ab OR 'sinoxal':ti,ab OR 'sr 96669':ti,ab OR 'sr96669':ti,ab OR 'transplastin':ti,ab OR 'velminox':ti,ab OR 'xaliplat':ti,ab OR 'xoplan':ti,ab))

19743

10781


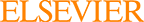


© 2020 Elsevier Life Sciences IP Limited except certain content provided by third parties.

Embase is a trade mark of Elsevier Life Sciences IP Limited. RELX Group and the RE symbol are trade marks of RELX Group plc, used under license.

2417

borderline:ti,ab,kw AND ('pancreas cancer'/exp OR (((pancreatic OR pancreas) NEAR/3 (cancer OR neoplasm* OR carcinoma* OR adenocarcinoma* OR adenoma* OR tumor* OR tumour*)):ti,ab))

#1

### Brought to you by [UZH Hauptbibliothek / Zentralbibliothek Zürich](http://www.uzh.ch/services/libraries.html)

[Elsevier logo Scopus](https://www.scopus.com/home.uri?zone=header&origin) [Search](https://www.scopus.com/search/form.uri?zone=TopNavBar&origin=searchadvanced&display=advanced) [Sources](https://www.scopus.com/sources.uri?zone=TopNavBar&origin=searchadvanced) [Lists](https://www.scopus.com/results/storedList.uri?listId=myDocList&origin=searchadvanced&zone=TopNavBar)

[SciVal ↗](https://www.scival.com/home)

[Alerts](https://www.scopus.com/signin.uri?&origin=searchadvanced&zone=TopNavBar)

[Sign in](https://www.scopus.com/signin.uri?origin=searchadvanced&zone=TopNavBar)

Advanced search

Help

Institu


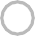

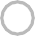

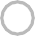

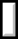

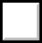

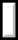

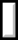

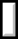

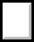

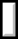


[Create account](https://www.scopus.com/signin.uri?origin=searchadvanced&zone=TopNavBar)

[Compare sources](https://www.scopus.com/source/eval/handle.uri?navbar=t&origin=searchadvanced&zone=TopNavBar) [▻](https://www.scopus.com/source/eval/handle.uri?navbar=t&origin=searchadvanced&zone=TopNavBar)

### Documents Authors Affiliations Advanced

OR

+

AND NOT

+

[Search tips ▻](https://www.scopus.com/standard/help.uri?topic=11365&anchor=tips)

### Operators

Enter query string

AND

+

### TITLE-ABS-KEY("abi 007" OR "abi007" OR "abraxane" OR "albumin bound paclitaxel" OR "albumin-bound paclitaxel" OR "anzatax" OR "apealea" OR "asotax" OR "biotax" OR "bms 181339" OR "bms181339" OR "bmy 45622" OR "bmy45622" OR "bristaxol" OR "britaxol" OR "coroxane" OR "dts 301" OR "dts301" OR "endotag-1" OR "formoxol" OR "genexol" OR "genexol pm" OR "hunxol" OR "ifaxol" OR "infinnium" OR "intaxel" OR "mbt 0206" OR "mbt0206" OR "medixel" OR "mitotax" OR "nab paclitaxel" OR "nanoparticle albumin bound paclitaxel" OR "nsc 125973" OR "nsc 673089" OR "nsc125973" OR "nsc673089" OR "oas pac 100" OR "oaspac100" OR "oncogel" OR "onxol" OR "pacitaxel" OR "paclitaxel" OR "paclitaxel nab" OR "pacxel" OR "padexol" OR "parexel" OR "paxceed" OR "paxene" OR "paxus" OR "pazenir" OR "praxel" OR "sb 05 (terpenoid)" OR "sb05 (terpenoid)" OR "taxocris" OR "taxol" OR "taxus (drug)" OR "taycovit" OR "yewtaxan") AND TITLE-ABS-KEY("2` deoxy 2`, 2` difluorocytidine" OR "2`, 2` difluorodeoxycytidine" OR "d 07001" OR "d07001" OR "difluorodeoxycytidine" OR "ff 10832" OR "ff10832" OR "gembin" OR "gemci- cell" OR "gemcisela" OR "gemcitabin" OR "gemcitabina" OR "gemcitabine" OR "gemcitabine hydrochloride" OR "gemcitabinum" OR "gemcite" OR "gemcitina" OR "gemcitom" OR "gemedac" OR "gemkabi" OR "gemliquid (drug)" OR "gemsol (drug)" OR "gemstad" OR "gemstada" OR "gemtro" OR "gemzar" OR "getmisi" OR "gitrabin" OR "infugem" OR "ly 188011" OR "ly188011" OR

Authors ▻

Biological Entities ▻

Chemical Entities ▻

Conferences ▻

Document ▻

Editors ▻

Funding ▻

Field codes [▻](https://www.scopus.com/standard/help.uri?topic=11236&anchor=tips)

PRE/

+

W/

+

Textual Content ▻

Affiliations ▻

|  |
| --- |
|  |
|  |
|  |
|  |
|  |
|  |
|  |
|  |

"ribozar")

[Add Author name / Affiliation](#_bookmark0) [Clear form](#_bookmark0)

Keywords ▻

Publication ▻

References ▻

Subject Areas ▻

Outline query

|  |
| --- |
|  |
|  |
|  |

ALL("Cognitive architectures") AND AUTHOR-NAME(smith)

TITLE-ABS-KEY(*somatic complaint wom?n) AND PUBYEAR AFT 1993 SRCTITLE(*field ornith*) AND VOLUME(75) AND ISSUE(1) AND PAGES(53-66)

### Search history [▻](https://www.scopus.com/standard/help.uri?topic=11227&origin=searchhistory&anchor=anchor)

Combine queries... *e.g. #1 AND NOT #3*

( TITLE-ABS-KEY ( borderline ) AND TITLE-ABS-KEY ( ( pancreatic OR pancreas ) W/3 ( cancer OR neoplasm* OR carcinoma* OR adenocarcinoma* OR adenoma* OR tumor* OR tumour* ) ) ) AND ( ( TITLE-ABS-KEY ( folfirinox ) OR ( TITLE-ABS-KEY ( "5 formyl 5, 6, 7, 8 tetrahydrofolic acid" OR "5 formyltetrahydrofolate" OR "5 formyltetrahydrofolic acid" OR "5 formyltetrahydropteroylglutamic acid" OR "folinic acid" OR "folinic acid sf" OR "folinic acid sf rescue" OR "formyltetrahydrofolate" OR "formyltetrahydrofolates" OR "formyltetrahydropteroylglutamic acid" OR "lencovorin" OR "leucovorin" OR "leukovoran" OR "leukovorin" OR "n formyltetrahydrofolic acid" OR "n5 formyl 5, 6, 7, 8 tetrahydropteroylglutamic acid" OR "n5 formyl tetrahydrofolic acid" OR "n5 formyltetrahydrofolate" OR "n5 formyltetrahydrofolic acid" ) AND TITLE-ABS-KEY ( "2, 4 dioxo 5 fluoropyrimidine" OR "5 fluoro 2, 4 pyrimidinedione" OR "5 fluoro uracil" OR "5 fluoropyrimidine 2, 4 dione" OR "5 fluorouracil" OR "5 fluoruracil" OR "5 fu" OR "accusite" OR "actino- hermal" OR "adrucil" OR "agicil" OR "carac" OR "cinkef u" OR "cinkef-u" OR "effluderm" OR "efudex" OR "efudix" OR "efurix" OR "eurofluor (drug)" OR "f6627" OR "fivoflu" OR "fluoro uracil" OR "fluoroblastin" OR "fluoroplex" OR "fluorouracil" OR "fluorouracil 5" OR "fluorouracil sodium" OR "fluorouracile" OR "fluorouracilo" OR "fluoruracil" OR "fluouracil" OR "fluoxan" OR "flurablastin" OR "fluracedyl" OR "fluracil" OR "fluracilium" OR "fluril" OR "fluro uracil" OR "fluroblastin" OR "fluroblastine" OR "ifacil" OR "nsc 18913" OR "nsc 19893" OR

"nsc18913" OR "nsc19893" OR "oncofu" OR "ribofluor" OR "ro 2 9757" OR "ro 2- 9757" OR "ro2 9757" OR "ro2-9757" OR "tolak" OR "uflahex" OR "uraciflor" OR "utoral" ) AND TITLE-ABS-KEY ( "7 ethyl 10 (4 piperidinopiperidinocarbonyloxy) camptothecin" OR "calmtop" OR "campto" OR "camptosar" OR "camptothecin, 7

ethyl 10 (4 piperidinopiperidinocarbonyloxy)" OR "cpt 11" OR "cpt11" OR "irinotecan" OR "irinotecan hydrochloride" OR "irinotecan hydrochloride trihydrate" OR "irinotel" OR "topotecin" ) AND TITLE-ABS-KEY ( "axiplatin" OR "bendaplatin" OR "crisapla" OR "croloxat" OR "dacotin" OR "dacplat" OR "ebeoxal" OR "elatofen" OR "eloxatin" OR "eloxatine" OR "elplat" OR "euroxaliplatin" OR "geneplatin" OR "gessedil" OR "heloxatin" OR "lipoxal" OR "mbp 426" OR

5 "mbp426" OR "medoxa" OR "oksaliplatin" OR "oksaliplatina" OR "oplat" OR

"oxalato 1, 2 cyclohexanediamine platinum" OR "oxalatoplatinum" OR "oxalatplatin"

OR "oxali" OR "oxalip" OR "oxaliplan" OR "oxaliplatin" OR "oxaliplatina" OR "oxaliplatine" OR "oxaliplatino" OR "oxaliplatinum" OR "oxaliprol" OR "oxaliquid" OR "oxalisan" OR "oxalisin" OR "oxalizor" OR "oxaltic" OR "oxaltina" OR "oxamed (oxaliplatin)" OR "oxaplamyl" OR "oxaviatin" OR "platinum 1, 2 cyclohexanediamine oxalate" OR "platinum 1, 2 diaminocyclohexane oxalate" OR "platinum oxalate 1, 2 diaminocyclohexane" OR "platinum trans (oxalato) (1, 2 diaminocyclohexane)" OR "platox" OR "plaxitin" OR "rectoxal" OR "riboxatin" OR "rp 54780" OR "rp54780" OR "sinoxal" OR "sr 96669" OR "sr96669" OR

"transplastin" OR "velminox" OR "xaliplat" OR "xoplan" ) ) ) OR ( TITLE-ABS-

KEY ( "abi 007" OR "abi007" OR "abraxane" OR "albumin bound paclitaxel" OR "albumin-bound paclitaxel" OR "anzatax" OR "apealea" OR "asotax" OR "biotax" OR "bms 181339" OR "bms181339" OR "bmy 45622" OR "bmy45622" OR

"bristaxol" OR "britaxol" OR "coroxane" OR "dts 301" OR "dts301" OR "endotag- 1" OR "formoxol" OR "genexol" OR "genexol pm" OR "hunxol" OR "ifaxol" OR "infinnium" OR "intaxel" OR "mbt 0206" OR "mbt0206" OR "medixel" OR "mitotax" OR "nab paclitaxel" OR "nanoparticle albumin bound paclitaxel" OR "nsc 125973" OR "nsc 673089" OR "nsc125973" OR "nsc673089" OR "oas pac 100"

OR "oaspac100" OR "oncogel" OR "onxol" OR "pacitaxel" OR "paclitaxel" OR "paclitaxel nab" OR "pacxel" OR "padexol" OR "parexel" OR "paxceed" OR "paxene" OR "paxus" OR "pazenir" OR "praxel" OR "sb 05 (terpenoid)" OR "sb05 (terpenoid)" OR "taxocris" OR "taxol" OR "taxus (drug)" OR "taycovit" OR "yewtaxan" ) AND TITLE-ABS-KEY ( "2` deoxy 2`, 2` difluorocytidine" OR "2`, 2` difluorodeoxycytidine" OR "d 07001" OR "d07001" OR "difluorodeoxycytidine" OR "ff 10832" OR "ff10832" OR "gembin" OR "gemci-cell" OR "gemcisela" OR "gemcitabin" OR "gemcitabina" OR "gemcitabine" OR "gemcitabine hydrochloride" OR "gemcitabinum" OR "gemcite" OR "gemcitina" OR "gemcitom" OR "gemedac" OR "gemkabi" OR "gemliquid (drug)" OR "gemsol (drug)" OR "gemstad" OR "gemstada" OR "gemtro" OR "gemzar" OR "getmisi" OR "gitrabin" OR "infugem" OR "ly 188011" OR "ly188011" OR "ribozar" ) ) ) AND ( LIMIT-TO ( LANGUAGE ,

"English" ) )

View Less ▻

[245 document results](https://www.scopus.com/search/history/results.uri?origin=searchhistory&shid=5) [▻](#_bookmark0) [▻](https://www.scopus.com/search/history/edit.uri?shid=5) [▻](https://www.scopus.com/search/history/delete.uri?shid=5)


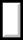


▻

( TITLE-ABS-KEY ( borderline ) AND TITLE-ABS-KEY ( ( pancreatic OR pancreas ) W/3 ( cancer OR neoplasm* OR carcinoma* OR adenocarcinoma* OR adenoma* OR


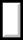
tumor* OR tumour* ) ) ) AND ( ( TITLE-ABS-KEY ( folfirinox ) OR ( TITLE-ABS-KEY ( "5 formyl 5, 6, 7, 8 tetrahydrofolic acid" OR "5 formyltetrahydrofolate" OR "5 formyltetrahydrofolic acid" OR "5 formyltetrahydropteroylglutamic acid" OR "folinic acid" OR "folinic acid sf" OR "folinic acid sf rescue" OR "formyltetrahydrofolate" OR "formyltetrahydrofolates" OR "formyltetrahydropteroylglutamic acid" OR "lencovorin" OR "leucovorin" OR "leukovoran" OR "leukovorin" OR "n formyltetrahydrofolic acid" OR "n5 formyl 5, 6, 7, 8 tetrahydropteroylglutamic acid" OR "n5 formyl tetrahydrofolic acid" OR "n5 formyltetrahydrofolate" OR "n5 formyltetrahydrofolic acid" ) AND TITLE-ABS-KEY ( "2, 4 dioxo 5 fluoropyrimidine" OR "5 fluoro 2, 4 pyrimidinedione" OR "5 fluoro uracil" OR "5 fluoropyrimidine 2, 4 dione" OR "5 fluorouracil" OR "5 fluoruracil" OR "5 fu" OR "accusite" OR "actino- hermal" OR "adrucil" OR "agicil" OR "carac" OR "cinkef u" OR "cinkef-u" OR "effluderm" OR "efudex" OR "efudix" OR "efurix" OR "eurofluor (drug)" OR "f6627" OR "fivoflu" OR "fluoro uracil" OR "fluoroblastin" OR "fluoroplex" OR "fluorouracil" OR "fluorouracil 5" OR "fluorouracil sodium" OR "fluorouracile" OR "fluorouracilo" OR "fluoruracil" OR "fluouracil" OR "fluoxan" OR "flurablastin" OR "fluracedyl" OR "fluracil" OR "fluracilium" OR "fluril" OR "fluro uracil" OR "fluroblastin" OR "fluroblastine" OR "ifacil" OR "nsc 18913" OR "nsc 19893" OR

▻

"nsc18913" OR "nsc19893" OR "oncofu" OR "ribofluor" OR "ro 2 9757" OR "ro 2- 9757" OR "ro2 9757" OR "ro2-9757" OR "tolak" OR "uflahex" OR "uraciflor" OR "utoral" ) AND TITLE-ABS-KEY ( "7 ethyl 10 (4 piperidinopiperidinocarbonyloxy) camptothecin" OR "calmtop" OR "campto" OR "camptosar" OR "camptothecin, 7 ethyl 10 (4 piperidinopiperidinocarbonyloxy)" OR "cpt 11" OR "cpt11" OR "irinotecan" OR "irinotecan hydrochloride" OR "irinotecan hydrochloride trihydrate" OR "irinotel" OR "topotecin" ) AND TITLE-ABS-KEY ( "axiplatin" OR "bendaplatin" OR "crisapla" OR "croloxat" OR "dacotin" OR "dacplat" OR "ebeoxal" OR "elatofen" OR "eloxatin" OR "eloxatine" OR "elplat" OR "euroxaliplatin" OR "geneplatin" OR "gessedil" OR "heloxatin" OR "lipoxal" OR "mbp 426" OR

4 "mbp426" OR "medoxa" OR "oksaliplatin" OR "oksaliplatina" OR "oplat" OR "oxalato 1, 2 cyclohexanediamine platinum" OR "oxalatoplatinum" OR "oxalatplatin" OR "oxali" OR "oxalip" OR "oxaliplan" OR "oxaliplatin" OR "oxaliplatina" OR "oxaliplatine" OR "oxaliplatino" OR "oxaliplatinum" OR "oxaliprol" OR "oxaliquid" OR "oxalisan" OR "oxalisin" OR "oxalizor" OR "oxaltic" OR "oxaltina" OR "oxamed (oxaliplatin)" OR "oxaplamyl" OR "oxaviatin" OR "platinum 1, 2 cyclohexanediamine oxalate" OR "platinum 1, 2 diaminocyclohexane oxalate" OR "platinum oxalate 1, 2 diaminocyclohexane" OR "platinum trans (oxalato) (1, 2 diaminocyclohexane)" OR "platox" OR "plaxitin" OR "rectoxal" OR "riboxatin" OR "rp 54780" OR "rp54780" OR "sinoxal" OR "sr 96669" OR "sr96669" OR "transplastin" OR "velminox" OR "xaliplat" OR "xoplan" ) ) ) OR ( TITLE-ABS-

KEY ( "abi 007" OR "abi007" OR "abraxane" OR "albumin bound paclitaxel" OR "albumin-bound paclitaxel" OR "anzatax" OR "apealea" OR "asotax" OR "biotax" OR "bms 181339" OR "bms181339" OR "bmy 45622" OR "bmy45622" OR

[263 document results](https://www.scopus.com/search/history/results.uri?origin=searchhistory&shid=4) [▻](#_bookmark0) [▻](https://www.scopus.com/search/history/edit.uri?shid=4) [▻](https://www.scopus.com/search/history/delete.uri?shid=4)

"bristaxol" OR "britaxol" OR "coroxane" OR "dts 301" OR "dts301" OR "endotag- 1" OR "formoxol" OR "genexol" OR "genexol pm" OR "hunxol" OR "ifaxol" OR "infinnium" OR "intaxel" OR "mbt 0206" OR "mbt0206" OR "medixel" OR "mitotax" OR "nab paclitaxel" OR "nanoparticle albumin bound paclitaxel" OR "nsc 125973" OR "nsc 673089" OR "nsc125973" OR "nsc673089" OR "oas pac 100"

OR "oaspac100" OR "oncogel" OR "onxol" OR "pacitaxel" OR "paclitaxel" OR "paclitaxel nab" OR "pacxel" OR "padexol" OR "parexel" OR "paxceed" OR "paxene" OR "paxus" OR "pazenir" OR "praxel" OR "sb 05 (terpenoid)" OR "sb05 (terpenoid)" OR "taxocris" OR "taxol" OR "taxus (drug)" OR "taycovit" OR "yewtaxan" ) AND TITLE-ABS-KEY ( "2` deoxy 2`, 2` difluorocytidine" OR "2`, 2` difluorodeoxycytidine" OR "d 07001" OR "d07001" OR "difluorodeoxycytidine" OR "ff 10832" OR "ff10832" OR "gembin" OR "gemci-cell" OR "gemcisela" OR "gemcitabin" OR "gemcitabina" OR "gemcitabine" OR "gemcitabine hydrochloride" OR "gemcitabinum" OR "gemcite" OR "gemcitina" OR "gemcitom" OR "gemedac" OR "gemkabi" OR "gemliquid (drug)" OR "gemsol (drug)" OR "gemstad" OR "gemstada" OR "gemtro" OR "gemzar" OR "getmisi" OR "gitrabin" OR "infugem" OR "ly 188011" OR "ly188011" OR "ribozar" ) ) )

View Less ▻

TITLE-ABS-KEY ( "abi 007" OR "abi007" OR "abraxane" OR "albumin bound paclitaxel" OR "albumin-bound paclitaxel" OR "anzatax" OR "apealea" OR "asotax" OR "biotax" OR "bms 181339" OR "bms181339" OR "bmy 45622" OR "bmy45622" OR "bristaxol" OR "britaxol" OR "coroxane" OR "dts 301" OR "dts301" OR "endotag-1" OR "formoxol" OR "genexol" OR "genexol pm" OR "hunxol" OR "ifaxol" OR "infinnium" OR "intaxel" OR "mbt 0206" OR "mbt0206" OR "medixel" OR "mitotax" OR "nab paclitaxel" OR "nanoparticle albumin bound paclitaxel" OR "nsc 125973" OR "nsc 673089" OR "nsc125973" OR "nsc673089" OR "oas pac 100" OR "oaspac100" OR "oncogel" OR "onxol" OR "pacitaxel" OR

3 "paclitaxel" OR "paclitaxel nab" OR "pacxel" OR "padexol" OR "parexel" OR

"paxceed" OR "paxene" OR "paxus" OR "pazenir" OR "praxel" OR "sb 05

(terpenoid)" OR "sb05 (terpenoid)" OR "taxocris" OR "taxol" OR "taxus (drug)" OR "taycovit" OR "yewtaxan" ) AND TITLE-ABS-KEY ( "2` deoxy 2`, 2` difluorocytidine" OR "2`, 2` difluorodeoxycytidine" OR "d 07001" OR "d07001" OR "difluorodeoxycytidine" OR "ff 10832" OR "ff10832" OR "gembin" OR "gemci-cell" OR "gemcisela" OR "gemcitabin" OR "gemcitabina" OR "gemcitabine" OR "gemcitabine hydrochloride" OR "gemcitabinum" OR "gemcite" OR "gemcitina" OR "gemcitom" OR "gemedac" OR "gemkabi" OR "gemliquid (drug)" OR "gemsol (drug)" OR "gemstad" OR "gemstada" OR "gemtro" OR "gemzar" OR "getmisi" OR "gitrabin" OR "infugem" OR "ly 188011" OR "ly188011" OR "ribozar" )

View Less ▻

[17,039 document results](https://www.scopus.com/search/history/results.uri?origin=searchhistory&shid=3) [▻](#_bookmark0) [▻](https://www.scopus.com/search/history/edit.uri?shid=3) [▻](https://www.scopus.com/search/history/delete.uri?shid=3)


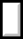


▻

TITLE-ABS-KEY ( folfirinox ) OR ( TITLE-ABS-KEY ( "5 formyl 5, 6, 7, 8 tetrahydrofolic

acid" OR "5 formyltetrahydrofolate" OR "5 formyltetrahydrofolic acid" OR "5 formyltetrahydropteroylglutamic acid" OR "folinic acid" OR "folinic acid sf" OR "folinic acid sf rescue" OR "formyltetrahydrofolate" OR "formyltetrahydrofolates" OR "formyltetrahydropteroylglutamic acid" OR "lencovorin" OR "leucovorin" OR "leukovoran" OR "leukovorin" OR "n formyltetrahydrofolic acid" OR "n5 formyl 5, 6, 7, 8 tetrahydropteroylglutamic acid" OR "n5 formyl tetrahydrofolic acid" OR "n5 formyltetrahydrofolate" OR "n5 formyltetrahydrofolic acid" ) AND TITLE-ABS-KEY ( "2, 4 dioxo 5 fluoropyrimidine" OR "5 fluoro 2, 4 pyrimidinedione" OR "5 fluoro uracil"

OR "5 fluoropyrimidine 2, 4 dione" OR "5 fluorouracil" OR "5 fluoruracil" OR "5 fu" OR "accusite" OR "actino-hermal" OR "adrucil" OR "agicil" OR "carac" OR "cinkef u" OR "cinkef-u" OR "effluderm" OR "efudex" OR "efudix" OR "efurix" OR "eurofluor (drug)" OR "f6627" OR "fivoflu" OR "fluoro uracil" OR "fluoroblastin" OR "fluoroplex" OR "fluorouracil" OR "fluorouracil 5" OR "fluorouracil sodium" OR "fluorouracile" OR "fluorouracilo" OR "fluoruracil" OR "fluouracil" OR "fluoxan" OR "flurablastin" OR "fluracedyl" OR "fluracil" OR "fluracilium" OR "fluril" OR "fluro uracil" OR "fluroblastin" OR "fluroblastine" OR "ifacil" OR "nsc 18913" OR "nsc 19893" OR "nsc18913" OR "nsc19893" OR "oncofu" OR "ribofluor" OR "ro 2

2 9757" OR "ro 2-9757" OR "ro2 9757" OR "ro2-9757" OR "tolak" OR "uflahex" OR

"uraciflor" OR "utoral" ) AND TITLE-ABS-KEY ( "7 ethyl 10 (4

piperidinopiperidinocarbonyloxy) camptothecin" OR "calmtop" OR "campto" OR "camptosar" OR "camptothecin, 7 ethyl 10 (4 piperidinopiperidinocarbonyloxy)" OR "cpt 11" OR "cpt11" OR "irinotecan" OR "irinotecan hydrochloride" OR "irinotecan hydrochloride trihydrate" OR "irinotel" OR "topotecin" ) AND TITLE-ABS-

KEY ( "axiplatin" OR "bendaplatin" OR "crisapla" OR "croloxat" OR "dacotin" OR "dacplat" OR "ebeoxal" OR "elatofen" OR "eloxatin" OR "eloxatine" OR "elplat" OR "euroxaliplatin" OR "geneplatin" OR "gessedil" OR "heloxatin" OR "lipoxal" OR "mbp 426" OR "mbp426" OR "medoxa" OR "oksaliplatin" OR "oksaliplatina" OR "oplat" OR "oxalato 1, 2 cyclohexanediamine platinum" OR "oxalatoplatinum" OR "oxalatplatin" OR "oxali" OR "oxalip" OR "oxaliplan" OR "oxaliplatin" OR "oxaliplatina" OR "oxaliplatine" OR "oxaliplatino" OR "oxaliplatinum" OR "oxaliprol" OR "oxaliquid" OR "oxalisan" OR "oxalisin" OR "oxalizor" OR "oxaltic" OR "oxaltina" OR "oxamed (oxaliplatin)" OR "oxaplamyl" OR "oxaviatin" OR "platinum 1, 2 cyclohexanediamine oxalate" OR "platinum 1, 2 diaminocyclohexane oxalate" OR "platinum oxalate 1, 2 diaminocyclohexane" OR "platinum trans (oxalato) (1, 2 diaminocyclohexane)" OR "platox" OR "plaxitin" OR "rectoxal" OR "riboxatin" OR "rp 54780" OR "rp54780" OR "sinoxal" OR "sr 96669" OR "sr96669" OR "transplastin" OR "velminox" OR "xaliplat" OR "xoplan" ) )

View Less ▻

TITLE-ABS-KEY ( borderline ) AND TITLE-ABS-KEY ( ( pancreatic OR pancreas ) W/3

1 ( cancer OR neoplasm* OR carcinoma* OR adenocarcinoma* OR adenoma* OR

tumor* OR tumour* ) )

[9,209 document results](https://www.scopus.com/search/history/results.uri?origin=searchhistory&shid=2)
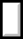
[▻](#_bookmark0) [▻](https://www.scopus.com/search/history/edit.uri?shid=2) [▻](https://www.scopus.com/search/history/delete.uri?shid=2)


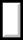
[1,344 document results](https://www.scopus.com/search/history/results.uri?origin=searchhistory&shid=1) [▻](#_bookmark0) [▻](https://www.scopus.com/search/history/edit.uri?shid=1) [▻](https://www.scopus.com/search/history/delete.uri?shid=1)

▻

▻

[Help improve Scopus](http://eepurl.com/cfBFzf)

[□ Top of page](#_bookmark0)

### About Scopus

[What is Scopus](https://www.elsevier.com/online-tools/scopus) [Content coverage](https://www.elsevier.com/online-tools/scopus/content-overview/) [Scopus blog](https://blog.scopus.com/) [Scopus API](https://dev.elsevier.com/) [Privacy matters](https://www.elsevier.com/about/our-business/policies/privacy-principles)

### Language

[日本語に切り替える](https://www.scopus.com/personalization/switch/Japanese.uri?origin&zone=footer&locale=ja_JP)[切换到简体中文](https://www.scopus.com/personalization/switch/Chinese.uri?origin&zone=footer&locale=zh_CN)

[切換到繁體中文](https://www.scopus.com/personalization/switch/Chinese.uri?origin&zone=footer&locale=zh_TW)

[Русский язык](https://www.scopus.com/personalization/switch/Russian.uri?origin&zone=footer&locale=ru_RU)

### Customer Service

[Help](https://www.scopus.com/standard/contactUs.uri?pageOrigin=footer) [Contact us](https://www.scopus.com/standard/contactForm.uri?pageOrigin=footer)

[Terms and conditions ↗](https://www.elsevier.com/locate/termsandconditions) [Privacy policy ↗](https://www.elsevier.com/locate/privacypolicy)

Copyright © [Elsevier B.V ↗](https://www.elsevier.com/). All rights reserved. Scopus® is a registered trademark of Elsevier B.V.

We use cookies to help provide and enhance our service and tailor content. By continuing, you agree to the [use of cookies](https://www.scopus.com/cookies/policy.uri).

□ [](#_bookmark1)

Cookies

Our site uses cookies to improve your experience. You can find out more about our use of cookies in About Cookies, including instructions on how to turn off cookies if you wish to do so. By continuing to browse this site you agree to us using cookies as described in [About Cookies](http://olabout.wiley.com/WileyCDA/Section/id-813473.html).


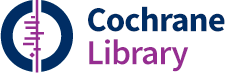
[I accept](#_bookmark1)

[**The Cochrane Library**](https://www.cochranelibrary.com/en/)

*Trusted evidence. Informed decisions. Better health.*

[](#_bookmark1)

[Open menu](#_bookmark1)

Access provided by: **UZH Hauptbibliothek / Zentralbibliothek Zürich**

#
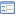
Scolaris Language Selector Scolaris Language Selector

[□ English  English](#_bookmark1)

[□ Sign In](https://www.cochranelibrary.com/c/portal/login?p_l_id=20907&redirect=%2Fadvanced-search%2Fsearch-manager)

Access provided by: **UZH Hauptbibliothek / Zentralbibliothek Zürich**

[Cochrane Reviews](#_bookmark1)

[Search Reviews (CDSR)](https://www.cochranelibrary.com/en/cdsr/reviews) [Browse Reviews](https://www.cochranelibrary.com/en/cdsr/reviews/topics)

[Issues](https://www.cochranelibrary.com/en/cdsr/table-of-contents) [Editorials](https://www.cochranelibrary.com/en/cdsr/editorials)

[Special Collections](https://www.cochranelibrary.com/en/special-collections) [Supplements](https://www.cochranelibrary.com/en/cdsr/supplements) [About the CDSR](https://www.cochranelibrary.com/en/cdsr/about-cdsr)

[Trials](#_bookmark1)

[Search Trials (CENTRAL)](https://www.cochranelibrary.com/en/central) [About CENTRAL](https://www.cochranelibrary.com/en/central/about-central)

[Clinical Answers](#_bookmark1)

[Browse Clinical Answers](https://www.cochranelibrary.com/en/cca) [About Clinical Answers](https://www.cochranelibrary.com/en/cca/about)

[About](#_bookmark1)

[About the Cochrane Library](https://www.cochranelibrary.com/en/about/about-cochrane-library) [About Cochrane Reviews](https://www.cochranelibrary.com/en/about/about-cochrane-reviews)

[About Cochrane Review Groups](https://www.cochranelibrary.com/en/about/cochrane-review-groups) [Information for authors](https://www.cochranelibrary.com/en/about/author-information)

[What's new](https://www.cochranelibrary.com/en/about/releases) [Help](#_bookmark1)

[Cochrane Library Training](https://www.wiley.com/network/cochranelibrarytraining) [Access options](https://www.cochranelibrary.com/en/help/access) [Permissions and reprints](https://www.cochranelibrary.com/en/help/permissions) [Media information](https://www.cochranelibrary.com/en/help/media) [Contact us](https://www.cochranelibrary.com/en/help/contact-us)

[Terms and conditions](https://www.cochranelibrary.com/en/about/terms-and-conditions) [Known issues](https://cochranesupport.wiley.com/s/article/cochrane-library-known-issues)

[About Cochrane](https://www.cochrane.org/about-us)



Explore new Cochrane Library features [**here**](https://www.cochranelibrary.com/about/releases).

[](#_bookmark1)

#
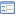
Scolaris Content Language Banner Portlet Scolaris Content Language Banner Portlet



We noticed your browser language is German.

You can select your preferred language at the top of any page, and you will see translated Cochrane Review sections in this language. Change to [German.](https://www.cochranelibrary.com/en/advanced-search/search-manager?p_p_id=scolariscontentlanguagebanner_WAR_scolarislanguageportlet&p_p_lifecycle=2&p_p_state=normal&p_p_mode=view&p_p_resource_id=set-content-language&p_p_cacheability=cacheLevelPage&_scolariscontentlanguagebanner_WAR_scolarislanguageportlet_contentLanguage=de)

[](#_bookmark1)

#
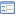
Scolaris Search Manager Portlet Scolaris Search Manager Portlet

**Advanced Search**

Search manager [Search](https://www.cochranelibrary.com/en/advanced-search)

[Search manager](https://www.cochranelibrary.com/en/advanced-search/search-manager) [Medical terms (MeSH)](https://www.cochranelibrary.com/en/advanced-search/mesh) [PICO searchBETA](https://www.cochranelibrary.com/en/advanced-search/pico)

□ *Search help*

□ *View saved searches*

□ *Save this search* □

Print

□


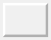


Print


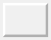
1.
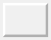


Save as

Save

Save as

□

□

Save


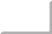


#1

borderline:ti,ab,kw AND ((pancreatic OR pancreas) NEAR/3 (cancer OR neoplasm* OR carcinoma* OR adenocarcinoma* OR adenoma* OR tumor* OR tumour*)):ti,ab,kw

borderline:ti,ab,kw AND ((pancreatic OR pancreas) NEAR/3 (cancer OR neoplasm* OR

S

#1

S □

MeSH □

Limits

MeSH

Limits

163

Cancel

Continue


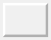
2.
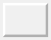


163

Cancel

Continue

□

□


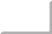


#2

folfirinox:ti,ab,kw OR (("5 formyl 5, 6, 7, 8 tetrahydrofolic acid" OR "5 formyltetrahydrofolate" OR "5 formyltetrahydrofolic acid" OR "5 formyltetrahydropteroylglutamic acid" OR "folinic acid" OR "folinic acid sf" OR "folinic acid sf rescue" OR "formyltetrahydrofolate" OR "formyltetrahydrofolates" OR "formyltetrahydropteroylglutamic acid" OR "lencovorin" OR "leucovorin" OR "leukovoran" OR

#2

"leukovorin" OR "n formyltetrahydrofolic acid" OR "n5 formyl 5, 6, 7, 8 tetrahydropteroylglutamic acid" OR "n5 formyl tetrahydrofolic acid" OR "n5 formyltetrahydrofolate" OR "n5 formyltetrahydrofolic acid"):ti,ab,kw AND ("2, 4 dioxo 5 fluoropyrimidine" OR "5 fluoro 2, 4 pyrimidinedione" OR "5 fluoro

uracil" OR "5 fluoropyrimidine 2, 4 dione" OR "5 fluorouracil" OR "5 fluoruracil" OR "5 fu" OR "accusite" OR "actino-hermal" OR "adrucil" OR "agicil" OR "carac" OR "cinkef u" OR "cinkef-u" OR "effluderm" OR "efudex" OR "efudix" OR "efurix" OR "eurofluor (drug)" OR "f6627" OR "fivoflu" OR "fluoro uracil" OR "fluoroblastin" OR "fluoroplex" OR "fluorouracil" OR "fluorouracil 5" OR "fluorouracil sodium" OR "fluorouracile" OR "fluorouracilo" OR "fluoruracil" OR "fluouracil" OR "fluoxan" OR "flurablastin" OR "fluracedyl" OR "fluracil" OR "fluracilium" OR "fluril" OR "fluro uracil" OR "fluroblastin" OR "fluroblastine" OR "ifacil" OR "nsc 18913" OR "nsc 19893" OR "nsc18913" OR "nsc19893" OR "oncofu" OR "ribofluor" OR "ro 2 9757" OR "ro 2-9757" OR "ro2 9757" OR "ro2-9757" OR "tolak" OR "uflahex" OR "uraciflor" OR "utoral"):ti,ab,kw AND ("7 ethyl 10 (4 piperidinopiperidinocarbonyloxy) camptothecin" OR "calmtop" OR "campto" OR "camptosar" OR "camptothecin, 7 ethyl 10 (4 piperidinopiperidinocarbonyloxy)" OR "cpt 11" OR "cpt11" OR "irinotecan" OR "irinotecan hydrochloride" OR "irinotecan hydrochloride trihydrate" OR "irinotel" OR "topotecin"):ti,ab,kw AND ("axiplatin" OR "bendaplatin" OR "crisapla" OR "croloxat" OR "dacotin" OR "dacplat" OR "ebeoxal" OR "elatofen" OR "eloxatin" OR "eloxatine" OR "elplat" OR "euroxaliplatin" OR "geneplatin" OR "gessedil" OR "heloxatin" OR "lipoxal" OR "mbp 426" OR "mbp426" OR "medoxa" OR "oksaliplatin" OR "oksaliplatina" OR "oplat" OR "oxalato 1, 2 cyclohexanediamine platinum" OR "oxalatoplatinum" OR "oxalatplatin" OR "oxali" OR "oxalip" OR "oxaliplan" OR "oxaliplatin" OR "oxaliplatina" OR "oxaliplatine" OR "oxaliplatino" OR "oxaliplatinum" OR "oxaliprol" OR "oxaliquid" OR "oxalisan" OR "oxalisin" OR "oxalizor" OR "oxaltic" OR "oxaltina" OR "oxamed (oxaliplatin)" OR "oxaplamyl" OR "oxaviatin" OR "platinum 1, 2 cyclohexanediamine oxalate" OR "platinum 1, 2 diaminocyclohexane oxalate" OR "platinum oxalate 1, 2 diaminocyclohexane" OR "platinum trans (oxalato) (1, 2 diaminocyclohexane)" OR "platox" OR "plaxitin" OR "rectoxal" OR "riboxatin" OR "rp 54780" OR "rp54780" OR "sinoxal" OR "sr 96669" OR "sr96669" OR "transplastin" OR "velminox" OR "xaliplat" OR "xoplan"):ti,ab,kw)

folfirinox:ti,ab,kw OR (("5 formyl 5, 6, 7, 8 tetrahydrofolic acid" OR "5

S

S □

MeSH □

MeSH

Limits

752

Cancel

Continue


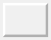
3.
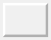


Limits

752

Cancel

Continue

□

□


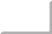


#3

("abi 007" OR "abi007" OR "abraxane" OR "albumin bound paclitaxel" OR "albumin-bound paclitaxel" OR "anzatax" OR "apealea" OR "asotax" OR "biotax" OR "bms 181339" OR "bms181339" OR "bmy 45622" OR "bmy45622" OR "bristaxol" OR "britaxol" OR "coroxane" OR "dts 301" OR "dts301" OR "endotag-1" OR "formoxol" OR "genexol" OR "genexol pm" OR "hunxol" OR "ifaxol" OR "infinnium" OR "intaxel" OR "mbt 0206" OR "mbt0206" OR "medixel" OR "mitotax" OR "nab paclitaxel" OR "nanoparticle albumin bound paclitaxel" OR "nsc 125973" OR "nsc 673089" OR "nsc125973" OR "nsc673089" OR "oas pac 100" OR "oaspac100" OR "oncogel" OR "onxol" OR "pacitaxel" OR "paclitaxel" OR "paclitaxel nab" OR "pacxel" OR "padexol" OR "parexel" OR "paxceed" OR "paxene" OR "paxus" OR "pazenir" OR "praxel" OR "sb 05 (terpenoid)" OR "sb05 (terpenoid)" OR "taxocris" OR "taxol" OR "taxus (drug)" OR "taycovit" OR "yewtaxan"):ti,ab,kw AND ("2` deoxy 2`, 2` difluorocytidine" OR "2`, 2` difluorodeoxycytidine" OR "d 07001" OR "d07001" OR "difluorodeoxycytidine" OR "ff 10832" OR "ff10832" OR "gembin" OR "gemci-cell" OR "gemcisela" OR "gemcitabin" OR "gemcitabina" OR "gemcitabine" OR "gemcitabine hydrochloride" OR "gemcitabinum" OR "gemcite" OR "gemcitina" OR "gemcitom" OR "gemedac" OR "gemkabi" OR "gemliquid (drug)" OR "gemsol (drug)" OR "gemstad" OR "gemstada" OR "gemtro" OR "gemzar" OR "getmisi" OR "gitrabin" OR "infugem" OR "ly 188011" OR "ly188011" OR "ribozar"):ti,ab,kw

#3

S □

("abi 007" OR "abi007" OR "abraxane" OR "albumin bound paclitaxel" OR "albumin-bound

S

MeSH

Limits

1368

Cancel

Continue


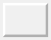
4.
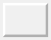


MeSH □

Limits

1368

Cancel

Continue

□

□


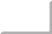


#4

#1 AND (#2 OR #3)

#1 AND (#2 OR #3)

S

#4

S □

MeSH □

MeSH

Limits


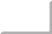


65

Cancel

Continue


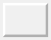
5.
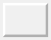


Limits

65

Cancel

Continue

□

□

#5

S □

MeSH □


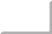


#5

Type a search term or use the S

S

MeSH

Limits

N/A

Cancel

Continue

Highlight orphan lines

□ *Search help*

□ *View saved searches*

□ *Save this search* □

Print

Limits

N/A

Cancel

Continue

□ Clear all

Print

# Scolaris Search Results Portlet


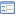
**Scolaris Search Results Portlet**

Save

Save as

Save

Save as

**Filter your results**  

Year 

Year first published 0 [2020](#_bookmark1)

8 [2019](#_bookmark1)

10 [2018](#_bookmark1)

14 [2017](#_bookmark1)

15 [2016](#_bookmark1)

Custom Range:

to

Apply

Clear

Apply

Clear

yyyy

yyyy

Date 

Date added to CENTRAL trials database 1 [The last 3 months](#_bookmark1)

2 [The last 6 months](#_bookmark1)

7 [The last 9 months](#_bookmark1) 10 [The last year](#_bookmark1)

44 [The last 2 years](#_bookmark1)

Custom Range:

to

Apply

Clear

Apply

Clear

dd/mm/yyyy

dd/mm/yyyy

Source 

32 [Embase](#_bookmark1)

20 [ICTRP](#_bookmark1)

12 [CT.gov](#_bookmark1)

3 [PubMed](#_bookmark1)

[Cochrane Reviews 0](https://www.cochranelibrary.com/en/advanced-search/search-manager?searchBy=-1&searchText&isWordVariations&resultPerPage=25&searchType=searchManager&forceTypeSelection=true&selectedType=review&displayText&orderBy=relevancy&p_p_id=scolarissearchresultsportlet_WAR_scolarissearchresults&p_p_lifecycle=0&p_p_state=normal&p_p_mode=view&p_p_col_id=column-1&p_p_col_pos=1&p_p_col_count=2) [Cochrane Protocols 0](https://www.cochranelibrary.com/en/advanced-search/search-manager?searchBy=-1&searchText&isWordVariations&resultPerPage=25&searchType=searchManager&forceTypeSelection=true&selectedType=protocol&displayText&orderBy=relevancy&p_p_id=scolarissearchresultsportlet_WAR_scolarissearchresults&p_p_lifecycle=0&p_p_state=normal&p_p_mode=view&p_p_col_id=column-1&p_p_col_pos=1&p_p_col_count=2) [Trials 65](https://www.cochranelibrary.com/en/advanced-search/search-manager?searchBy=-1&searchText&isWordVariations&resultPerPage=25&searchType=searchManager&forceTypeSelection=true&selectedType=central&displayText&orderBy=relevancy&p_p_id=scolarissearchresultsportlet_WAR_scolarissearchresults&p_p_lifecycle=0&p_p_state=normal&p_p_mode=view&p_p_col_id=column-1&p_p_col_pos=1&p_p_col_count=2)

[Editorials 0](https://www.cochranelibrary.com/en/advanced-search/search-manager?searchBy=-1&searchText&isWordVariations&resultPerPage=25&searchType=searchManager&forceTypeSelection=true&selectedType=editorial&displayText&orderBy=relevancy&p_p_id=scolarissearchresultsportlet_WAR_scolarissearchresults&p_p_lifecycle=0&p_p_state=normal&p_p_mode=view&p_p_col_id=column-1&p_p_col_pos=1&p_p_col_count=2)

[Special collections 0](https://www.cochranelibrary.com/en/advanced-search/search-manager?searchBy=-1&searchText&isWordVariations&resultPerPage=25&searchType=searchManager&forceTypeSelection=true&selectedType=specialcollections&displayText&orderBy=displayDate-true&p_p_id=scolarissearchresultsportlet_WAR_scolarissearchresults&p_p_lifecycle=0&p_p_state=normal&p_p_mode=view&p_p_col_id=column-1&p_p_col_pos=1&p_p_col_count=2) [Clinical Answers 0](https://www.cochranelibrary.com/en/advanced-search/search-manager?searchBy=-1&searchText&isWordVariations&resultPerPage=25&searchType=searchManager&forceTypeSelection=true&selectedType=cca&displayText&orderBy=relevancy&p_p_id=scolarissearchresultsportlet_WAR_scolarissearchresults&p_p_lifecycle=0&p_p_state=normal&p_p_mode=view&p_p_col_id=column-1&p_p_col_pos=1&p_p_col_count=2) Other Reviews

# 65 Trials matching "#4 - #1 AND (#2 OR #3)"

[*Cochran*](https://www.cochranelibrary.com/)*e* [Central Register of Controlled Trials](https://www.cochranelibrary.com/) Issue 5 of 12, May 2020

Deselect all (65) Order by

Export selected citation(s)

Relevancy

Export selected citation(s)

Relevancy

Relevancy

1

## [Phase II study of modified FOLFIRINO](https://www.cochranelibrary.com/central/doi/10.1002/central/CN-01865355/full)X [as](https://www.cochranelibrary.com/central/doi/10.1002/central/CN-01865355/full)

Results per page

25

25

25

## [neoadjuvant chemotherapy for borderlin](https://www.cochranelibrary.com/central/doi/10.1002/central/CN-01865355/full)e [resectable](https://www.cochranelibrary.com/central/doi/10.1002/central/CN-01865355/full) [pancreatic cance](https://www.cochranelibrary.com/central/doi/10.1002/central/CN-01865355/full)r [and locally advanced unresectable](https://www.cochranelibrary.com/central/doi/10.1002/central/CN-01865355/full) [pancreatic cancer](https://www.cochranelibrary.com/central/doi/10.1002/central/CN-01865355/full)

JPRN-UMIN000015707

[http://www.who.int/trialsearch/Trial2.aspx?TrialID=JPRN-UMIN000015707,](http://www.who.int/trialsearch/Trial2.aspx?TrialID=JPRN-UMIN000015707)

**2014** | added to CENTRAL: 31 March 2019 | 2019 Issue 3 ICTRP

2

## [Neoadjuvant FOLFIRINO](https://www.cochranelibrary.com/central/doi/10.1002/central/CN-01556788/full)X [or Nab-paclitaxe](https://www.cochranelibrary.com/central/doi/10.1002/central/CN-01556788/full)l [With](https://www.cochranelibrary.com/central/doi/10.1002/central/CN-01556788/full) [Gemcitabin](https://www.cochranelibrary.com/central/doi/10.1002/central/CN-01556788/full)e [for Borderlin](https://www.cochranelibrary.com/central/doi/10.1002/central/CN-01556788/full)e [Resectable Pancreatic Cancer](https://www.cochranelibrary.com/central/doi/10.1002/central/CN-01556788/full)

NCT02717091

https://clinicaltrials.gov/show/NCT02717091, **2016** | added to CENTRAL: 31 May 2018 | 2018 Issue 5

CT.gov

3

## [Phase II study of neoadjuvant FOLFIRINO](https://www.cochranelibrary.com/central/doi/10.1002/central/CN-01875886/full)X [or](https://www.cochranelibrary.com/central/doi/10.1002/central/CN-01875886/full)

[**nab-paclitaxe**](https://www.cochranelibrary.com/central/doi/10.1002/central/CN-01875886/full)**l** [**with gemcitabin**](https://www.cochranelibrary.com/central/doi/10.1002/central/CN-01875886/full)**e** [**for borderlin**](https://www.cochranelibrary.com/central/doi/10.1002/central/CN-01875886/full)**e** [**resectable**](https://www.cochranelibrary.com/central/doi/10.1002/central/CN-01875886/full) [**pancreatic cancer**](https://www.cochranelibrary.com/central/doi/10.1002/central/CN-01875886/full)

JPRN-UMIN000017718

[http://www.who.int/trialsearch/Trial2.aspx?TrialID=JPRN-UMIN000017718,](http://www.who.int/trialsearch/Trial2.aspx?TrialID=JPRN-UMIN000017718)

**2015** | added to CENTRAL: 31 March 2019 | 2019 Issue 3 ICTRP

4

## [MFOLFIRINOX And Stereotactic Radiotherapy (SBRT) for](https://www.cochranelibrary.com/central/doi/10.1002/central/CN-01991873/full) [Pancreatic Cance](https://www.cochranelibrary.com/central/doi/10.1002/central/CN-01991873/full)r [With High Risk and Locally Advanced](https://www.cochranelibrary.com/central/doi/10.1002/central/CN-01991873/full) [Disease](https://www.cochranelibrary.com/central/doi/10.1002/central/CN-01991873/full)

NCT04089150

https://clinicaltrials.gov/show/NCT04089150, **2019** | added to CENTRAL: 31 October 2019 | 2019 Issue 10

CT.gov

5

## [Feasibility and efficacy of gemcitabin](https://www.cochranelibrary.com/central/doi/10.1002/central/CN-01839563/full)e [plus albumin-bound](https://www.cochranelibrary.com/central/doi/10.1002/central/CN-01839563/full) [paclitaxe](https://www.cochranelibrary.com/central/doi/10.1002/central/CN-01839563/full)l [combination therapy (GEM+nabPTX therapy) in](https://www.cochranelibrary.com/central/doi/10.1002/central/CN-01839563/full) [patients with borderlin](https://www.cochranelibrary.com/central/doi/10.1002/central/CN-01839563/full)e [resectable pancreatic cance](https://www.cochranelibrary.com/central/doi/10.1002/central/CN-01839563/full)r [in the](https://www.cochranelibrary.com/central/doi/10.1002/central/CN-01839563/full) [neoadjvant setting](https://www.cochranelibrary.com/central/doi/10.1002/central/CN-01839563/full)

JPRN-UMIN000022000

[http://www.who.int/trialsearch/Trial2.aspx?TrialID=JPRN-UMIN000022000,](http://www.who.int/trialsearch/Trial2.aspx?TrialID=JPRN-UMIN000022000)

**2016** | added to CENTRAL: 31 March 2019 | 2019 Issue 3 ICTRP

6

[**Feasibility study of neoadjuvant gemcitabine plus**](https://www.cochranelibrary.com/central/doi/10.1002/central/CN-01827971/full)

[**nab-paclitaxe**](https://www.cochranelibrary.com/central/doi/10.1002/central/CN-01827971/full)**l** [**for borderlin**](https://www.cochranelibrary.com/central/doi/10.1002/central/CN-01827971/full)**e** [**resectable pancreatic cancer**](https://www.cochranelibrary.com/central/doi/10.1002/central/CN-01827971/full)

JPRN-UMIN000023591

[http://www.who.int/trialsearch/Trial2.aspx?TrialID=JPRN-UMIN000023591,](http://www.who.int/trialsearch/Trial2.aspx?TrialID=JPRN-UMIN000023591)

**2016** | added to CENTRAL: 31 March 2019 | 2019 Issue 3 ICTRP

7

## [Combination of Anti-PD-1 Antibody and Chemotherapy in](https://www.cochranelibrary.com/central/doi/10.1002/central/CN-01983249/full) [Pancreatic Cancer](https://www.cochranelibrary.com/central/doi/10.1002/central/CN-01983249/full)

NCT03983057

https://clinicaltrials.gov/show/NCT03983057, **2019** | added to CENTRAL: 30 September 2019 | 2019 Issue 09

CT.gov

8

## [Gemcitabine-nab-paclitaxe](https://www.cochranelibrary.com/central/doi/10.1002/central/CN-01607794/full)l [as neoadjuvant treatment for](https://www.cochranelibrary.com/central/doi/10.1002/central/CN-01607794/full) [resectable and borderlin](https://www.cochranelibrary.com/central/doi/10.1002/central/CN-01607794/full)e [resectable pancreatic](https://www.cochranelibrary.com/central/doi/10.1002/central/CN-01607794/full) [adenocarcinoma](https://www.cochranelibrary.com/central/doi/10.1002/central/CN-01607794/full)

E Vicente, B Ielpo, E Vicente, Y Quijano, H Duran, R Caruso, E Diaz, I Fabra, L Malave, V Ferri, S Lazzaro, D Kalivaci, L Manino, A Zafra

HPB, **2017**, 19, S78‐ | added to CENTRAL: 30 June 2018 | 2018 Issue 6 Embase

9

## [Neoadjuvant chemoradiation therapy in borderlin](https://www.cochranelibrary.com/central/doi/10.1002/central/CN-01139517/full)e [resectable](https://www.cochranelibrary.com/central/doi/10.1002/central/CN-01139517/full) [pancreatic adenocarcinoma](https://www.cochranelibrary.com/central/doi/10.1002/central/CN-01139517/full)

S Shafi, A Kaubisch

Pancreas, **2015**, 44(8), 1413‐ | added to CENTRAL: 30 April 2016 | 2016 Issue

4

Embase 10

## [Gemcitabin](https://www.cochranelibrary.com/central/doi/10.1002/central/CN-01582140/full)e [+ Nab-paclitaxe](https://www.cochranelibrary.com/central/doi/10.1002/central/CN-01582140/full)l [With LDE-225 (Hedgehog](https://www.cochranelibrary.com/central/doi/10.1002/central/CN-01582140/full) [Inhibitor) as Neoadjuvant Therapy for Pancreatic](https://www.cochranelibrary.com/central/doi/10.1002/central/CN-01582140/full) [Adenocarcinoma](https://www.cochranelibrary.com/central/doi/10.1002/central/CN-01582140/full)

NCT01431794

https://clinicaltrials.gov/show/NCT01431794, **2011** | added to CENTRAL: 31 May 2018 | 2018 Issue 5

CT.gov

11

## [Neoadjuvant GMCI Plus mFOLFIRINOX and](https://www.cochranelibrary.com/central/doi/10.1002/central/CN-01591725/full) [Chemoradiation for Non-Metastatic Pancreatic](https://www.cochranelibrary.com/central/doi/10.1002/central/CN-01591725/full) [Adenocarcinoma](https://www.cochranelibrary.com/central/doi/10.1002/central/CN-01591725/full)

NCT02446093

https://clinicaltrials.gov/show/NCT02446093, **2015** | added to CENTRAL: 31 May 2018 | 2018 Issue 5

CT.gov 12

## [Neoadjuvant mFolfirinox With or Without Preoperative](https://www.cochranelibrary.com/central/doi/10.1002/central/CN-01595976/full) [Concomitant Chemoradiotherapy in Patients With Borderline](https://www.cochranelibrary.com/central/doi/10.1002/central/CN-01595976/full) [Resectable Pancreatic Carcinom](https://www.cochranelibrary.com/central/doi/10.1002/central/CN-01595976/full)a [(PANDAS-PRODIGE 44)](https://www.cochranelibrary.com/central/doi/10.1002/central/CN-01595976/full)

NCT02676349

https://clinicaltrials.gov/show/NCT02676349, **2016** | added to CENTRAL: 31 May 2018 | 2018 Issue 5

CT.gov

13

## [Phase I study of neoadjuvant chemotherapy of gemcitabine](https://www.cochranelibrary.com/central/doi/10.1002/central/CN-01880452/full) [plus nab-paclitaxe](https://www.cochranelibrary.com/central/doi/10.1002/central/CN-01880452/full)l [for patients with borderlin](https://www.cochranelibrary.com/central/doi/10.1002/central/CN-01880452/full)e [resectable](https://www.cochranelibrary.com/central/doi/10.1002/central/CN-01880452/full) [pancreatic cancer](https://www.cochranelibrary.com/central/doi/10.1002/central/CN-01880452/full)

JPRN-UMIN000018382

[http://www.who.int/trialsearch/Trial2.aspx?TrialID=JPRN-UMIN000018382,](http://www.who.int/trialsearch/Trial2.aspx?TrialID=JPRN-UMIN000018382)

**2015** | added to CENTRAL: 31 March 2019 | 2019 Issue 3 ICTRP

14

## [Correction: total Neoadjuvant Therapy With FOLFIRINOX](https://www.cochranelibrary.com/central/doi/10.1002/central/CN-01922530/full) [Followed by Individualized Chemoradiotherapyfor](https://www.cochranelibrary.com/central/doi/10.1002/central/CN-01922530/full) [BorderlineResectablePancreaticAdenocarcinoma: aPhase 2](https://www.cochranelibrary.com/central/doi/10.1002/central/CN-01922530/full) [Clinical Trial (JAMA Oncology (2018) 4: 7 (963-969) DOI:](https://www.cochranelibrary.com/central/doi/10.1002/central/CN-01922530/full) [10.1001/jamaoncol.2018.0329)](https://www.cochranelibrary.com/central/doi/10.1002/central/CN-01922530/full)

JAMA oncology, **2018**, 4(10), 1439 | added to CENTRAL: 30 April 2019 |

2019 Issue 04 Embase

15

## [De (kosten)effectiviteit van neoadjuvante FOLFIRINOX](https://www.cochranelibrary.com/central/doi/10.1002/central/CN-01906200/full) [versus neoadjuvante chemoradiotherapie met gemcitabin](https://www.cochranelibrary.com/central/doi/10.1002/central/CN-01906200/full)e [en](https://www.cochranelibrary.com/central/doi/10.1002/central/CN-01906200/full) [adjuvante gemcitabin](https://www.cochranelibrary.com/central/doi/10.1002/central/CN-01906200/full)e [voor patiënten met (borderline)](https://www.cochranelibrary.com/central/doi/10.1002/central/CN-01906200/full) [resectabel pancreaskanker - PREOPANC-2 studie](https://www.cochranelibrary.com/central/doi/10.1002/central/CN-01906200/full)

NTR7292

[http://www.who.int/trialsearch/Trial2.aspx?TrialID=NTR7292,](http://www.who.int/trialsearch/Trial2.aspx?TrialID=NTR7292) **2018** | added to CENTRAL: 31 March 2019 | 2019 Issue 3

ICTRP

16

## [Immunotherapy Study in Borderlin](https://www.cochranelibrary.com/central/doi/10.1002/central/CN-01578187/full)e [Resectable or Locally](https://www.cochranelibrary.com/central/doi/10.1002/central/CN-01578187/full) [Advanced Unresectable Pancreatic Cancer](https://www.cochranelibrary.com/central/doi/10.1002/central/CN-01578187/full)

NCT01836432

https://clinicaltrials.gov/show/NCT01836432, **2013** | added to CENTRAL: 31 May 2018 | 2018 Issue 5

CT.gov

17

## [Preoperative gemcitabin](https://www.cochranelibrary.com/central/doi/10.1002/central/CN-01802497/full)e [plus nab-paclitaxel, concurrent 3D](https://www.cochranelibrary.com/central/doi/10.1002/central/CN-01802497/full) [conformal radiation therapy for borderlin](https://www.cochranelibrary.com/central/doi/10.1002/central/CN-01802497/full)e [resectable](https://www.cochranelibrary.com/central/doi/10.1002/central/CN-01802497/full) [pancreatic cancer: phase I study](https://www.cochranelibrary.com/central/doi/10.1002/central/CN-01802497/full)

JPRN-UMIN000012456

[http://www.who.int/trialsearch/Trial2.aspx?TrialID=JPRN-UMIN000012456,](http://www.who.int/trialsearch/Trial2.aspx?TrialID=JPRN-UMIN000012456)

**2013** | added to CENTRAL: 31 March 2019 | 2019 Issue 3 ICTRP

18

## [Preoperative gemcitabin](https://www.cochranelibrary.com/central/doi/10.1002/central/CN-01888654/full)e [plus nab-paclitaxel, concurrent 3D](https://www.cochranelibrary.com/central/doi/10.1002/central/CN-01888654/full) [conformal radiation therapy for resectable and borderline](https://www.cochranelibrary.com/central/doi/10.1002/central/CN-01888654/full) [resectable pancreatic cancer: phase II study](https://www.cochranelibrary.com/central/doi/10.1002/central/CN-01888654/full)

JPRN-UMIN000027758

[http://www.who.int/trialsearch/Trial2.aspx?TrialID=JPRN-UMIN000027758,](http://www.who.int/trialsearch/Trial2.aspx?TrialID=JPRN-UMIN000027758)

**2017** | added to CENTRAL: 31 March 2019 | 2019 Issue 3 ICTRP

19

## [Phase II Neoadjuvant Chemotheraphy (Gemcitabin](https://www.cochranelibrary.com/central/doi/10.1002/central/CN-01548732/full)e [and](https://www.cochranelibrary.com/central/doi/10.1002/central/CN-01548732/full) [Nab-Paclitaxe](https://www.cochranelibrary.com/central/doi/10.1002/central/CN-01548732/full)l [vs. mFOLFIRINOX) and Sterotatic Body](https://www.cochranelibrary.com/central/doi/10.1002/central/CN-01548732/full) [Radiation Therapy for Borderlin](https://www.cochranelibrary.com/central/doi/10.1002/central/CN-01548732/full)e [Resectable Pancreatic](https://www.cochranelibrary.com/central/doi/10.1002/central/CN-01548732/full) [Cancer](https://www.cochranelibrary.com/central/doi/10.1002/central/CN-01548732/full)

NCT02241551

https://clinicaltrials.gov/show/NCT02241551, **2014** | added to CENTRAL: 31 May 2018 | 2018 Issue 5

CT.gov

20

## [Randomized phase II/III study of gemcitabin](https://www.cochranelibrary.com/central/doi/10.1002/central/CN-01827309/full)e [and](https://www.cochranelibrary.com/central/doi/10.1002/central/CN-01827309/full) [nab-paclitaxe](https://www.cochranelibrary.com/central/doi/10.1002/central/CN-01827309/full)l [therapy versus S-1 and concurrent](https://www.cochranelibrary.com/central/doi/10.1002/central/CN-01827309/full) [radiotherapy as neoadjuvant treatment for Borderline](https://www.cochranelibrary.com/central/doi/10.1002/central/CN-01827309/full) [resectable pancreatic cancer](https://www.cochranelibrary.com/central/doi/10.1002/central/CN-01827309/full)

JPRN-UMIN000026858

[http://www.who.int/trialsearch/Trial2.aspx?TrialID=JPRN-UMIN000026858,](http://www.who.int/trialsearch/Trial2.aspx?TrialID=JPRN-UMIN000026858)

**2017** | added to CENTRAL: 31 March 2019 | 2019 Issue 3 ICTRP

21

## [Value of FDG PET/CT in early response assessment of](https://www.cochranelibrary.com/central/doi/10.1002/central/CN-01267620/full) [neoadjuvant chemotherapy for patients with pancreatic](https://www.cochranelibrary.com/central/doi/10.1002/central/CN-01267620/full) [adenocarcinoma](https://www.cochranelibrary.com/central/doi/10.1002/central/CN-01267620/full)

K Grant, J Miller, M Lotze, N Bahary, A Singhi, B Kurland, H Zeh, J Mountz Journal of nuclear medicine. Conference: society of nuclear medicine and molecular imaging annual meeting, SNMMI 2016. San diego, CA united states. Conference start: 20160611. Conference end: 20160615. Conference publication: (var.pagings), **2016**, 57(no pagination) | added to CENTRAL: 28 February 2017 | 2017 Issue 2

Embase

22

## [A phase II trial of neoadjuvant gemcitabine/nab-paclitaxe](https://www.cochranelibrary.com/central/doi/10.1002/central/CN-01793702/full)l [and](https://www.cochranelibrary.com/central/doi/10.1002/central/CN-01793702/full) [SBRT for potentially resectable pancreas cancer: an](https://www.cochranelibrary.com/central/doi/10.1002/central/CN-01793702/full) [evaluation of acute toxicity](https://www.cochranelibrary.com/central/doi/10.1002/central/CN-01793702/full)

M Palta, BG Czito, E Duffy, M Malicki, D Niedzwiecki, JL Abbruzzese, HE Uronis, GC Blobe, DG Blazer, C Willett

Journal of clinical oncology, **2018**, 36(15) | added to CENTRAL: 31 March 2019 | 2019 Issue 3

Embase

23

## [Neoadjuvant FOLFIRINO](https://www.cochranelibrary.com/central/doi/10.1002/central/CN-01988287/full)X [versus adjuvant gemcitabin](https://www.cochranelibrary.com/central/doi/10.1002/central/CN-01988287/full)e [in](https://www.cochranelibrary.com/central/doi/10.1002/central/CN-01988287/full) [pancreatic cancer](https://www.cochranelibrary.com/central/doi/10.1002/central/CN-01988287/full)

AR Wolfe, ED Miller, LI Abushahin, J Cloyd, A Manilchuck, M Dillhoff, DAD Pardo, AM Noonan, TM Williams

Journal of clinical oncology, **2019**, 37 | added to CENTRAL: 31 October 2019 | 2019 Issue 10

Embase

24

## [Comparisons of different neoadjuvant chemotherapy regimens](https://www.cochranelibrary.com/central/doi/10.1002/central/CN-01938398/full) [with or without stereotactic body radiation therapy for](https://www.cochranelibrary.com/central/doi/10.1002/central/CN-01938398/full) [borderlin](https://www.cochranelibrary.com/central/doi/10.1002/central/CN-01938398/full)e [resectable pancreatic cancer: study protocol of a](https://www.cochranelibrary.com/central/doi/10.1002/central/CN-01938398/full) [prospective, randomized phase II trial (BRPCNCC-1)](https://www.cochranelibrary.com/central/doi/10.1002/central/CN-01938398/full)

S Gao, X Zhu, X Shi, K Cao, Y Bian, H Jiang, K Wang, S Guo, H Zhang, G Jin

Radiation oncology (London, England), **2019**, 14(1), 52 | added to CENTRAL: 30 June 2019 | 2019 Issue 06

PubMed Embase

25

## [A phase II trial of Down staging Chemotherapy with](https://www.cochranelibrary.com/central/doi/10.1002/central/CN-01839242/full) [Nab-paclitaxe](https://www.cochranelibrary.com/central/doi/10.1002/central/CN-01839242/full)l [plus Gemcitabin](https://www.cochranelibrary.com/central/doi/10.1002/central/CN-01839242/full)e [in patients with locally](https://www.cochranelibrary.com/central/doi/10.1002/central/CN-01839242/full) [advanced pancreatic cancer](https://www.cochranelibrary.com/central/doi/10.1002/central/CN-01839242/full)

JPRN-UMIN000022241

[http://www.who.int/trialsearch/Trial2.aspx?TrialID=JPRN-UMIN000022241,](http://www.who.int/trialsearch/Trial2.aspx?TrialID=JPRN-UMIN000022241)

**2016** | added to CENTRAL: 31 March 2019 | 2019 Issue 3 ICTRP

[1](https://www.cochranelibrary.com/en/advanced-search/search-manager?min_year&max_year&custom_min_year&custom_max_year&searchBy=-1&searchText&selectedType=central&isWordVariations&resultPerPage=25&searchType=searchManager&orderBy=relevancy&publishDateTo&publishDateFrom&publishYearTo&publishYearFrom&displayText&forceTypeSelection=true&p_p_id=scolarissearchresultsportlet_WAR_scolarissearchresults&p_p_lifecycle=0&p_p_state=normal&p_p_mode=view&p_p_col_id=column-1&p_p_col_pos=1&p_p_col_count=2&cur=1)

[2](https://www.cochranelibrary.com/en/advanced-search/search-manager?min_year&max_year&custom_min_year&custom_max_year&searchBy=-1&searchText&selectedType=central&isWordVariations&resultPerPage=25&searchType=searchManager&orderBy=relevancy&publishDateTo&publishDateFrom&publishYearTo&publishYearFrom&displayText&forceTypeSelection=true&p_p_id=scolarissearchresultsportlet_WAR_scolarissearchresults&p_p_lifecycle=0&p_p_state=normal&p_p_mode=view&p_p_col_id=column-1&p_p_col_pos=1&p_p_col_count=2&cur=2)

[3](https://www.cochranelibrary.com/en/advanced-search/search-manager?min_year&max_year&custom_min_year&custom_max_year&searchBy=-1&searchText&selectedType=central&isWordVariations&resultPerPage=25&searchType=searchManager&orderBy=relevancy&publishDateTo&publishDateFrom&publishYearTo&publishYearFrom&displayText&forceTypeSelection=true&p_p_id=scolarissearchresultsportlet_WAR_scolarissearchresults&p_p_lifecycle=0&p_p_state=normal&p_p_mode=view&p_p_col_id=column-1&p_p_col_pos=1&p_p_col_count=2&cur=3)

[Next](https://www.cochranelibrary.com/en/advanced-search/search-manager?min_year&max_year&custom_min_year&custom_max_year&searchBy=-1&searchText&selectedType=central&isWordVariations&resultPerPage=25&searchType=searchManager&orderBy=relevancy&publishDateTo&publishDateFrom&publishYearTo&publishYearFrom&displayText&forceTypeSelection=true&p_p_id=scolarissearchresultsportlet_WAR_scolarissearchresults&p_p_lifecycle=0&p_p_state=normal&p_p_mode=view&p_p_col_id=column-1&p_p_col_pos=1&p_p_col_count=2&cur=2)

Cochrane


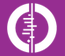

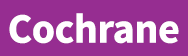


[About Cochrane](https://www.cochranelibrary.com/about-cochrane1)

[Cochrane.org](https://www.cochrane.org/evidence) [Who we are](https://www.cochrane.org/about-us) [Get involved](https://join.cochrane.org/)

[Consumer Network](https://consumers.cochrane.org/) [Partners](https://www.cochrane.org/about-us/our-funders-and-partners) [Colloquium](https://colloquium.cochrane.org/)

[In the news](https://www.cochrane.org/news) [Publications](https://www.cochranelibrary.com/publications)

[Cochrane Library](https://www.cochranelibrary.com/library)

[Library Homepage](https://www.cochranelibrary.com/) [Cochrane Reviews (CDSR)](https://www.cochranelibrary.com/advanced-search/cdsr/about-cdsr) [Trials (CENTRAL)](https://www.cochranelibrary.com/central) [Cochrane Clinical Answers](https://www.cochranelibrary.com/cca/about)

[Cochrane Library App](https://www.cochranelibrary.com/about/cochrane-library-app) [Journal Club](https://www.cochranelibrary.com/cdsr/journal-club)

[Podcasts](https://www.cochranelibrary.com/about/podcasts) [Community](https://www.cochranelibrary.com/community)

[Community](http://community.cochrane.org/) [Archie log-in](https://archie.cochrane.org/)

[Training and support](http://training.cochrane.org/) [Methods](http://methods.cochrane.org/)

[Software](https://community.cochrane.org/help)

[Jobs and opportunities](https://www.cochrane.org/news/jobs) [Contact Us](https://www.cochranelibrary.com/contact-us)

[General enquiries](http://www.cochrane.org/contact) [Cochrane Library support](https://www.cochranelibrary.com/help/contact-us) [Chief Executive Officer](https://community.cochrane.org/organizational-info/people/central-executive-team/chief-executive-officers-office/team) [Editor in Chief](http://community.cochrane.org/organizational-info/people/central-executive-team/editorial-methods/team)

[Cochrane groups](https://www.cochrane.org/about-us/our-global-community) [Media](http://www.cochrane.org/media) [Accessibility](https://www.cochranelibrary.com/accessibility)

[Browse Publications](https://onlinelibrary.wiley.com/) [Browse by Subject](https://onlinelibrary.wiley.com/)

[Advertisers & Agents](https://onlinelibrary.wiley.com/advertisers) [Contact Us](https://hub.wiley.com/community/support/cochrane-library)

[Help & Support](https://hub.wiley.com/community/support/cochrane-library) [Terms & Conditions](https://onlinelibrary.wiley.com/terms-and-conditions)

Copyright © 2000 - 2020 by [John Wiley & Sons, Inc.](http://www.wiley.com/) All Rights Reserved Review our [**Privacy Policy**](https://www.wiley.com/en-gb/privacy)


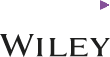


[close](#_bookmark1) [Close ](#_bookmark1)

[Review tools & navigation](#_bookmark1)

[□ Close menu ](#_bookmark1)

#
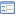
Scolaris Search Portlet Scolaris Search Portlet

Title Abstract Keyword

Title Abstract Keyword


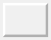
Title Abstract Keyword

Search

□

[Browse](https://www.cochranelibrary.com/en/browse-by-topic) [Advanced search](https://www.cochranelibrary.com/en/advanced-search) [Cochrane Reviews](#_bookmark1)

[Search Reviews (CDSR)](https://www.cochranelibrary.com/en/cdsr/reviews) [Browse Reviews](https://www.cochranelibrary.com/en/cdsr/reviews/topics)

[Issues](https://www.cochranelibrary.com/en/cdsr/table-of-contents) [Editorials](https://www.cochranelibrary.com/en/cdsr/editorials)

[Special Collections](https://www.cochranelibrary.com/en/special-collections) [Supplements](https://www.cochranelibrary.com/en/cdsr/supplements) [About the CDSR](https://www.cochranelibrary.com/en/cdsr/about-cdsr)

[Trials](#_bookmark1)

[Search Trials (CENTRAL)](https://www.cochranelibrary.com/en/central) [About CENTRAL](https://www.cochranelibrary.com/en/central/about-central)

[Clinical Answers](#_bookmark1)

[Browse Clinical Answers](https://www.cochranelibrary.com/en/cca) [About Clinical Answers](https://www.cochranelibrary.com/en/cca/about)

[About](#_bookmark1)

[About the Cochrane Library](https://www.cochranelibrary.com/en/about/about-cochrane-library) [About Cochrane Reviews](https://www.cochranelibrary.com/en/about/about-cochrane-reviews)

[About Cochrane Review Groups](https://www.cochranelibrary.com/en/about/cochrane-review-groups) [Information for authors](https://www.cochranelibrary.com/en/about/author-information)

[What's new](https://www.cochranelibrary.com/en/about/releases) [Help](#_bookmark1)

[Cochrane Library Training](https://www.wiley.com/network/cochranelibrarytraining) [Access options](https://www.cochranelibrary.com/en/help/access) [Permissions and reprints](https://www.cochranelibrary.com/en/help/permissions) [Media information](https://www.cochranelibrary.com/en/help/media) [Contact us](https://www.cochranelibrary.com/en/help/contact-us)

[Terms and conditions](https://www.cochranelibrary.com/en/about/terms-and-conditions) [Known issues](https://cochranesupport.wiley.com/s/article/cochrane-library-known-issues)

[About Cochrane](https://www.cochrane.org/about-us)

[Select your preferred language](#_bookmark1)


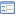
 [**Scolaris Language Selector Scolaris Language Selector**](#_bookmark1)

- [English  English](#_bookmark1)
- **Cochrane Review language**

Select your preferred language for Cochrane Reviews. You will see translated Review sections in your preferred language. Sections without translation will be in English.

English 

[Deutsc](https://www.cochranelibrary.com/en/advanced-search/search-manager?p_p_id=scolariscontentlanguagebanner_WAR_scolarislanguageportlet&p_p_lifecycle=2&p_p_state=normal&p_p_mode=view&p_p_resource_id=set-content-language&p_p_cacheability=cacheLevelPage&_scolariscontentlanguagebanner_WAR_scolarislanguageportlet_contentLanguage=de)h [Englis](https://www.cochranelibrary.com/en/advanced-search/search-manager?p_p_id=scolariscontentlanguagebanner_WAR_scolarislanguageportlet&p_p_lifecycle=2&p_p_state=normal&p_p_mode=view&p_p_resource_id=set-content-language&p_p_cacheability=cacheLevelPage&_scolariscontentlanguagebanner_WAR_scolarislanguageportlet_contentLanguage=en)h [Españo](https://www.cochranelibrary.com/en/advanced-search/search-manager?p_p_id=scolariscontentlanguagebanner_WAR_scolarislanguageportlet&p_p_lifecycle=2&p_p_state=normal&p_p_mode=view&p_p_resource_id=set-content-language&p_p_cacheability=cacheLevelPage&_scolariscontentlanguagebanner_WAR_scolarislanguageportlet_contentLanguage=es)l ف[ارسی](https://www.cochranelibrary.com/en/advanced-search/search-manager?p_p_id=scolariscontentlanguagebanner_WAR_scolarislanguageportlet&p_p_lifecycle=2&p_p_state=normal&p_p_mode=view&p_p_resource_id=set-content-language&p_p_cacheability=cacheLevelPage&_scolariscontentlanguagebanner_WAR_scolarislanguageportlet_contentLanguage=fa) [Françai](https://www.cochranelibrary.com/en/advanced-search/search-manager?p_p_id=scolariscontentlanguagebanner_WAR_scolarislanguageportlet&p_p_lifecycle=2&p_p_state=normal&p_p_mode=view&p_p_resource_id=set-content-language&p_p_cacheability=cacheLevelPage&_scolariscontentlanguagebanner_WAR_scolarislanguageportlet_contentLanguage=fr)s [Hrvatsk](https://www.cochranelibrary.com/en/advanced-search/search-manager?p_p_id=scolariscontentlanguagebanner_WAR_scolarislanguageportlet&p_p_lifecycle=2&p_p_state=normal&p_p_mode=view&p_p_resource_id=set-content-language&p_p_cacheability=cacheLevelPage&_scolariscontentlanguagebanner_WAR_scolarislanguageportlet_contentLanguage=hr)i [日本語](https://www.cochranelibrary.com/en/advanced-search/search-manager?p_p_id=scolariscontentlanguagebanner_WAR_scolarislanguageportlet&p_p_lifecycle=2&p_p_state=normal&p_p_mode=view&p_p_resource_id=set-content-language&p_p_cacheability=cacheLevelPage&_scolariscontentlanguagebanner_WAR_scolarislanguageportlet_contentLanguage=ja) [한국어](https://www.cochranelibrary.com/en/advanced-search/search-manager?p_p_id=scolariscontentlanguagebanner_WAR_scolarislanguageportlet&p_p_lifecycle=2&p_p_state=normal&p_p_mode=view&p_p_resource_id=set-content-language&p_p_cacheability=cacheLevelPage&_scolariscontentlanguagebanner_WAR_scolarislanguageportlet_contentLanguage=ko) [Bahasa Malaysi](https://www.cochranelibrary.com/en/advanced-search/search-manager?p_p_id=scolariscontentlanguagebanner_WAR_scolarislanguageportlet&p_p_lifecycle=2&p_p_state=normal&p_p_mode=view&p_p_resource_id=set-content-language&p_p_cacheability=cacheLevelPage&_scolariscontentlanguagebanner_WAR_scolarislanguageportlet_contentLanguage=ms)a [Polsk](https://www.cochranelibrary.com/en/advanced-search/search-manager?p_p_id=scolariscontentlanguagebanner_WAR_scolarislanguageportlet&p_p_lifecycle=2&p_p_state=normal&p_p_mode=view&p_p_resource_id=set-content-language&p_p_cacheability=cacheLevelPage&_scolariscontentlanguagebanner_WAR_scolarislanguageportlet_contentLanguage=pl)i [Português](https://www.cochranelibrary.com/en/advanced-search/search-manager?p_p_id=scolariscontentlanguagebanner_WAR_scolarislanguageportlet&p_p_lifecycle=2&p_p_state=normal&p_p_mode=view&p_p_resource_id=set-content-language&p_p_cacheability=cacheLevelPage&_scolariscontentlanguagebanner_WAR_scolarislanguageportlet_contentLanguage=pt)

[Русски](https://www.cochranelibrary.com/en/advanced-search/search-manager?p_p_id=scolariscontentlanguagebanner_WAR_scolarislanguageportlet&p_p_lifecycle=2&p_p_state=normal&p_p_mode=view&p_p_resource_id=set-content-language&p_p_cacheability=cacheLevelPage&_scolariscontentlanguagebanner_WAR_scolarislanguageportlet_contentLanguage=ru)й [தமிழ](https://www.cochranelibrary.com/en/advanced-search/search-manager?p_p_id=scolariscontentlanguagebanner_WAR_scolarislanguageportlet&p_p_lifecycle=2&p_p_state=normal&p_p_mode=view&p_p_resource_id=set-content-language&p_p_cacheability=cacheLevelPage&_scolariscontentlanguagebanner_WAR_scolarislanguageportlet_contentLanguage=ta)் [ภาษาไท](https://www.cochranelibrary.com/en/advanced-search/search-manager?p_p_id=scolariscontentlanguagebanner_WAR_scolarislanguageportlet&p_p_lifecycle=2&p_p_state=normal&p_p_mode=view&p_p_resource_id=set-content-language&p_p_cacheability=cacheLevelPage&_scolariscontentlanguagebanner_WAR_scolarislanguageportlet_contentLanguage=th)ย [繁體中文](https://www.cochranelibrary.com/en/advanced-search/search-manager?p_p_id=scolariscontentlanguagebanner_WAR_scolarislanguageportlet&p_p_lifecycle=2&p_p_state=normal&p_p_mode=view&p_p_resource_id=set-content-language&p_p_cacheability=cacheLevelPage&_scolariscontentlanguagebanner_WAR_scolarislanguageportlet_contentLanguage=zh_HANT) [简体中文](https://www.cochranelibrary.com/en/advanced-search/search-manager?p_p_id=scolariscontentlanguagebanner_WAR_scolarislanguageportlet&p_p_lifecycle=2&p_p_state=normal&p_p_mode=view&p_p_resource_id=set-content-language&p_p_cacheability=cacheLevelPage&_scolariscontentlanguagebanner_WAR_scolarislanguageportlet_contentLanguage=zh_HANS)

- **Website language**

Select your preferred language for the Cochrane Library website. English 

[Englis](https://www.cochranelibrary.com/en/advanced-search/search-manager)h [Español](https://www.cochranelibrary.com/es/advanced-search/search-manager)

[Sign In](https://www.cochranelibrary.com/c/portal/login?p_l_id=20907&redirect=%2Fadvanced-search%2Fsearch-manager)

[Visit Cochrane.org](http://www.cochrane.org/) PrevNext

May

2020

2020

May

**Su Mo Tu We Th Fr Sa**

[1](#_bookmark1) [2](#_bookmark1)

| [3](#_bookmark1) [4](#_bookmark1) | [5](#_bookmark1) | [6](#_bookmark1) | [7](#_bookmark1) | [8](#_bookmark1) [9](#_bookmark1) |
| --- | --- | --- | --- | --- |
| [10 11](#_bookmark1) | [12](#_bookmark1) | [13](#_bookmark1) | [14](#_bookmark1) | [15 16](#_bookmark1) |
| [17 18](#_bookmark1) | [19](#_bookmark1) | [20](#_bookmark1) | [21](#_bookmark1) | [22 23](#_bookmark1) |
| [24 25](#_bookmark1) | [26](#_bookmark1) | [27](#_bookmark1) | [28](#_bookmark1) | [29 30](#_bookmark1) |
| [31](#_bookmark1) |  |  |  |  |

Source: Use this to filter your results by the original source of the record.

[Save](https://www.cochranelibrary.com/en/advanced-search/search-manager?p_p_id=scolariscontentlanguagebanner_WAR_scolarislanguageportlet&p_p_lifecycle=2&p_p_state=normal&p_p_mode=view&p_p_resource_id=set-content-language&p_p_cacheability=cacheLevelPage)
